# Critical role of SMG7 in activation of the ATR-CHK1 axis in response to genotoxic stress

**DOI:** 10.1038/s41598-021-86957-x

**Published:** 2021-04-05

**Authors:** Kathleen Ho, Hongwei Luo, Wei Zhu, Yi Tang

**Affiliations:** 1 Department of Regenerative and Cancer Cell Biology, Albany Medical College, 47 New Scotland Ave, Albany, NY 12208 USA; 2 Key Laboratory of State Ethnic Affairs Commission for Biological Technology, College of Life Science, South-Central University for Nationalities, Wuhan, 430074 Hubei China

**Keywords:** Cancer, Cell biology, Molecular biology

## Abstract

CHK1 is a crucial DNA damage checkpoint kinase and its activation, which requires ATR and RAD17, leads to inhibition of DNA replication and cell cycle progression. Recently, we reported that SMG7 stabilizes and activates p53 to induce G_1_ arrest upon DNA damage; here we show that SMG7 plays a critical role in the activation of the ATR-CHK1 axis. Following genotoxic stress, *SMG7*-null cells exhibit deficient ATR signaling, indicated by the attenuated phosphorylation of CHK1 and RPA32, and importantly, unhindered DNA replication and fork progression. Through its 14-3-3 domain, SMG7 interacts directly with the Ser635-phosphorylated RAD17 and promotes chromatin retention of the 9-1-1 complex by the RAD17-RFC, an essential step to CHK1 activation. Furthermore, through maintenance of CHK1 activity, SMG7 controls G_2_-M transition and facilitates orderly cell cycle progression during recovery from replication stress. Taken together, our data reveals SMG7 as an indispensable signaling component in the ATR-CHK1 pathway during genotoxic stress response.

## Introduction

An essential function of the mammalian cell division cycle is to ensure that a cell can duplicate its genome accurately and efficiently during proliferation. This is a daunting task, as cells constantly encounter internal or external conditions that can hinder DNA replication or cause physical damage to DNA^1,2^. Unsurprisingly, cells employ conserved cell cycle checkpoint mechanisms, which help cope with DNA replication obstacles or damage, in order to maintain genome integrity^3^. The critical nature of these mechanisms are illustrated by the fact that loss or deficiency of their components can cause various human diseases associated with genome instability including cancer^2,4^.

The ataxia-telangiectasia mutated (ATM) and ATM- and Rad3-related (ATR) checkpoint kinases, which belong to the phosphatidylinositol 3-kinase-like kinase (PIKK) family, are two key regulators of the DNA damage response^3,5–8^. While ATM primarily functions in DNA double-strand break (DSB) signaling, ATR responds to various forms of DNA damage and replication stress caused by a wide range of genotoxic conditions^5,6^. Activated ATM and ATR phosphorylate and activate downstream effectors to halt cell cycle progression and DNA replication, so cells can repair damaged DNA or resolve any replication stress before entering the next phase of the cell cycle. For example, upon DNA damage (e.g., DSB), ATM activates the tumor suppressor p53, which can cause prolonged arrest of the cell cycle at the G_1_ phase^6^. CHK1 is a major downstream effector of ATR, and activation of the ATR-CHK1 axis leads to inhibition of DNA replication in S phase and G2 arrest^5^.

Activation of ATR is a complex and multi-step process that is initiated in response to stretches of replication protein A (RPA)-coated single-strand DNA (ssDNA)^5,9^. This aberrant DNA structure arises frequently at stalled replication forks when elongation and unwinding are uncoupled during replication, or at the DSB damage site following resection. The RPA-ssDNA complex serves a recruiting platform to bring together ATR and its activators—topoisomerase II binding protein 1 (TOPBP1)^10^ and Ewing tumor-associated antigen 1 (ETAA1)^11–13^. ATR does not bind RPA, and its recruitment is mediated by the direct interaction between its binding partner ATR-interacting protein (ATRIP) and RPA^14^. On the other hand, the recruitment of TOPBP1 occurs independently of ATR and relies on the 5′-ended ssDNA-double-stranded DNA (dsDNA) junction present in the stalled replication fork or resected DSB. Two additional components critical for TOPBP1 recruitment and subsequent ATR activation are the RAD17-RFC complex, which contains the checkpoint protein RAD17 and replication factor C subunits 2–5, and the RAD9-RAD1-HUS1 (9-1-1) checkpoint clamp^15–17^. Facilitated by RPA-ssDNA, RAD17-RFC loads to the 5′-ended ssDNA-dsDNA junction the 9-1-1 clamp complex, which further enables the recruitment of TOPBP1^18–23^. Unlike TOPBP1, ETAA1 binds RPA directly, and its recruitment appears to be independent on the 5′-ended ssDNA-dsDNA junction and the 9-1-1 complex^11–13^. Once brought together with ATR-ATRIP, TOPBP1 and ETAA1, both of which contain the ATR-activating domain (AAD), stimulate the kinase activity of ATR in the phosphorylation of its target substrates including downstream effectors (e.g., CHK1)^24,25^ as well as upstream signaling components.

One such signaling molecule is RAD17, a member of the AAA+ family of ATPases that is critical for 9-1-1 loading in activation of the ATR-CHK1 axis, and is phosphorylated on two serine residues S635 and S645 in the ATM/ATR SQ motif^26–28^. As reported in early studies, while ATM can mediate RAD17 phosphorylation, ATR is the major kinase for S635/645, and loss of these modifications impairs the RAD17 checkpoint function^27–29^. Importantly, phosphorylation of RAD17 on S635/645, which occurs in a cell cycle-dependent manner in undamaged cells, is required to maintain 9-1-1 at the DNA damage site and activate CHK1 in response to replication stress^28–30^. Interestingly, a recent study shows that RAD17 also regulates ATM activation in response to DSBs, involving phosphorylation of RAD17 on T622 by ATM^31^. Thus, these studies clearly demonstrate that RAD17 plays a key role in checkpoint regulation, and its phosphorylation by ATR and ATM is a pivotal signaling step in shaping the response of a cell to genotoxic stress.

Previously, we identified a critical role for the suppressor with morphological defects in genitalia 7 (SMG7) in the G1/S checkpoint in response to DNA damage response, through promoting ATM-mediated p53 stabilization and transcriptional activation^32^. Here, based on our initial observation that loss of SMG7 impairs CHK1 activation by ATR, we explored the idea that SMG7 may have a more general role in regulation of the genotoxic stress response. Interestingly, we found that the N-terminal 14-3-3 domain of SMG7 directly binds RAD17 and this interaction is completely dependent on ATR phosphorylation of RAD17 on S635. Sequence analysis revealed at the RAD17 C-terminal a conserved S635-containing SQ motif shared by other SMG7-binding partners including p53 and UPF1, indicating that the SMG7 14-3-3 domain may serve as a common protein-binding module in phospho-SQ-mediated signaling. Furthermore, we show that SMG7 constitutively associates with chromatin and promotes the recruitment of RAD9 to the DNA damage site. Importantly, unlike wild-type cells, the *SMG7*-deficient cells fail to inhibit S-phase DNA replication after genotoxic treatment, and their recovery from replication stress is significantly compromised because of the inability of cells to maintain CHK1 activity. Taken together, SMG7 has a critical function in activation of the ATR-CHK1 pathway, at least partly through engaging the RAD17-mediated DNA damage signaling.

## Results

### SMG7 is required for activation of the ATR-CHK1 pathway upon DNA damage

**Figure 1:**
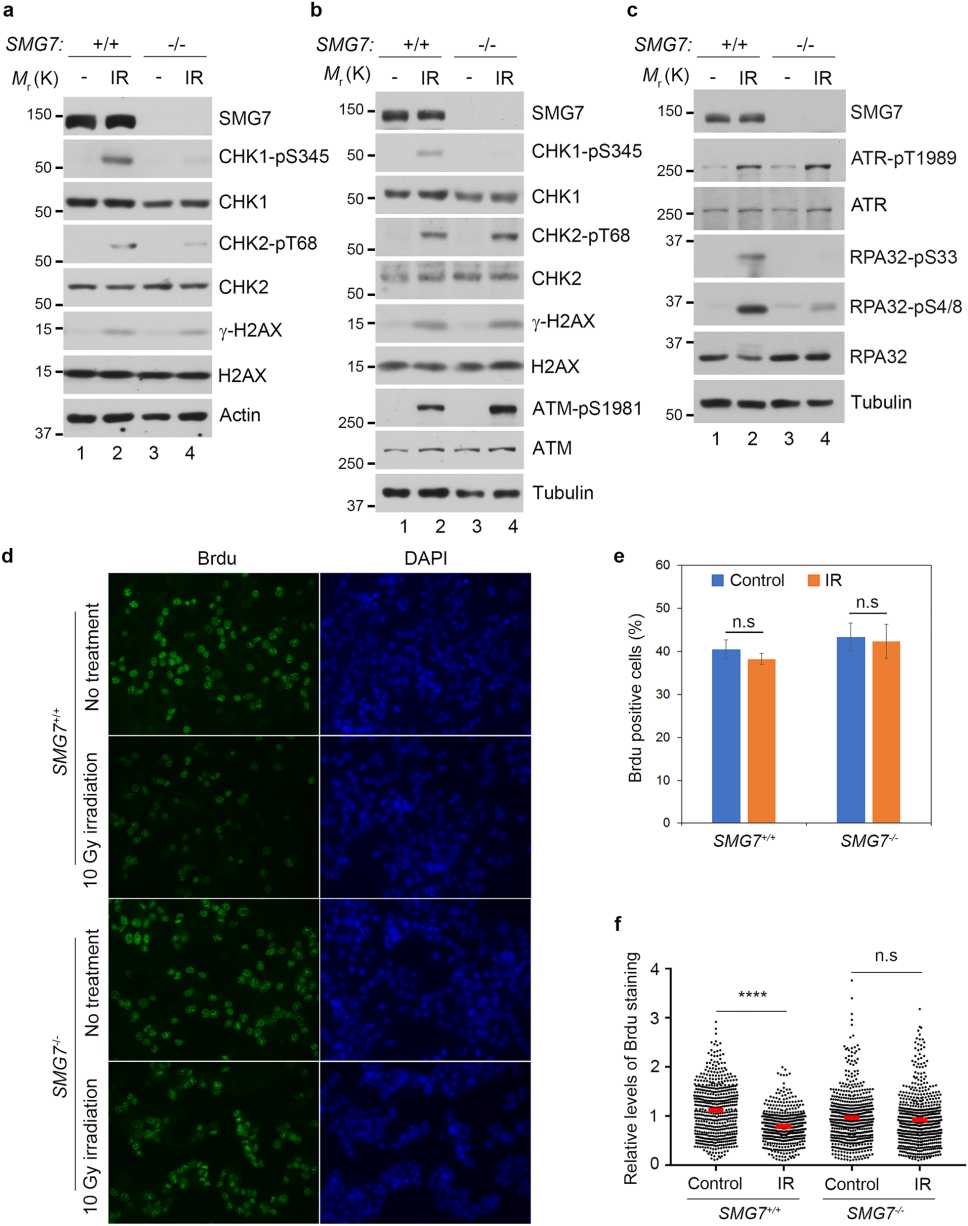
Loss of SMG7 impairs activation of CHK1 upon DNA damage. (**a**–**c**) Wild type and *SMG7*^*−/−*^ cells (**a** HCT116, **b**,**c** DLD1) were treated with ionizing radiation (**a**,**b** 10 Gy; **c** 20 Gy) and total cell extracts were examined for proteins in the ATM and ATR pathways by western blot analysis using antibodies as indicated including α-SMG7, α-CHK1-pS345, α-CHK1, α-CHK2-pT68, α-CHK2, α-γH2AX, α-H2AX, α-ATM-pS1981, α-ATM, α-ATR-pT1989, α-ATR, α-RPA32-pS4/8, α-RPA32-pS33, α-RPA32, α-Actin and α-Tubulin. Quantitation of relative levels of CHK1-pS345, CHK2-pT68, RPA-pS33, and RPA-pS4/8 in IR-treated cells are shown in Fig. S1a,d,e). (**d**) Wild type and *SMG7*^*−/−*^ HCT116 cells were treated with ionizing radiation (10 Gy, 1 h) and pulsed with 25 μM BrdU for 20 min, and analyzed by immunostaining using α-BrdU antibody (BU1/75). Representative images are shown. (**e**) Quantification of the percentage of BrdU-positive cells of the total population from (**d**). Data are presented as Mean ± SEM (n = 3 independent experiments) and were analyzed by ANOVA with Tukey post-test. n.s indicates not significant, *P* > 0.05. (**f**) Quantification of the relative intensities of BrdU staining in BrdU + cells from (**d**) using MetaMorph software. Red bars represent the mean intensities of each population. Data are shown as nuclear BrdU fluorescence intensities of cells pooled from three independent experiments and analyzed by ANOVA with Tukey post-test. **** indicates *P* < 0.001 and n.s, not significant, *P* > 0.05. Graphs were generated using GraphPad Prism.

SMG7 is known for its role in nonsense-mediated mRNA decay (NMD), a surveillance pathway that degrades premature stop codon-containing mRNA transcripts including those derived from alternative splicing such as the p53β isoform^32–34^. In our recent studies, we uncovered a novel function of SMG7 in stabilizing the tumor suppressor p53 by promoting ATM phosphorylation of MDM2^32^. Given that SMG7 contains the conserved 14-3-3 protein domain involved in numerous signaling pathways^35,36^, we reasoned that SMG7 likely has additional roles in the DNA damage response besides regulating the ATM-MDM2-p53 pathway. To explore this idea, we employed HCT116 *SMG7* knockout cells (*SMG7*^*−/−*^) generated in our previous studies and examined activation of CHK1 and CHK2, the two checkpoint kinases downstream of ATR and ATM, respectively^7^. As shown in Fig. 1a, following induction of DSBs (indicated by γ-H2AX) by ionizing radiation (IR), we observed similar levels of CHK2 phosphorylation on the ATM site T68 in both wild type and *SMG7*^*−/−*^ cells. Interestingly, loss of SMG7 caused a significant reduction of CHK1 phosphorylation on the ATR site S345—a well-established marker for CHK1 activation^24,25^ (Fig. 1a, lane 2 vs. lane 4 and Supplementary Fig. 1a), indicating that SMG7 has a specific role in activation of the ATR-CHK1 axis upon DNA damage. To corroborate this finding, we generated *SMG7* knockouts in human colorectal adenocarcinoma DLD1 cells using CRISPR-Cas9-mediated gene editing (Supplementary Fig. 1b and 1c). Notably, we made similar observations that deletion of *SMG7* impaired ATR-dependent CHK1 activation but had no effect on CHK2 (Fig. 1b, lane 2 vs. lane 4 and Supplementary Fig. 1d). Furthermore, we found that although wild type and *SMG7*^*−/−*^ cells exhibited minimal differences in the auto-phosphorylation of ATR on T1989, an ATR activation marker^37,38^, loss of SMG7 abrogated ATR-mediated phosphorylation of RPA32 on S33 (a direct ATR site) and S4/8 (the DNA-dependent protein kinase sites dependent on S33 phosphorylation)^39,40^ (Fig. 1c, lane 2 vs. lane 4 and Supplementary Fig. 1e and 1f). Thus, these results show that SMG7 is required for activation of the ATR-CHK1 axis and phosphorylation of some other ATR signaling components such as RPA32 but is dispensable for the intrinsic activity of ATR.

It is well known that the ATR-CHK1 pathway plays a key role in regulation of replication stress, and activation of CHK1 upon DNA damage inhibits DNA replication in S phase^41,42^. To assess the downstream effects of SMG7 on CHK1, we examined DNA synthesis in *SMG7*^*−/−*^ cells after IR. To this end, we exposed HCT116 cells to 10 Gy radiation and labeled them with BrdU to identify replicating cells, which comprise approximately 40% of the total population regardless of *SMG7* status (Fig. 1d,e). As expected, IR had no effect on the percentage of S-phase cells (Fig. 1e), but significantly reduced the intensity of BrdU staining in wild type cells (Fig. 1d,f). The lack of reduction of BrdU staining in *SMG7*^*−/−*^ cells indicates that DNA synthesis proceeded without hindrance despite DNA damage (Fig. 1f), consistent with impaired CHK1 activation in the absence of SMG7. We made similar observations in DLD1 cells (Supplementary Fig. 1g), which express mutant p53^43,44^, and importantly, re-introduction of ectopic SMG7 restored CHK1-pS345 phosphorylation and strongly inhibited DNA replication (Supplementary Fig. 1h,i). Taken together, these data indicate that SMG7 is critically important for regulation of the genotoxic stress response through activation of the ATR/CHK1 pathway—a function that is separate from p53-mediated cell cycle arrest following DNA damage.

### RAD17 is a binding partner of SMG7

**Figure 2:**
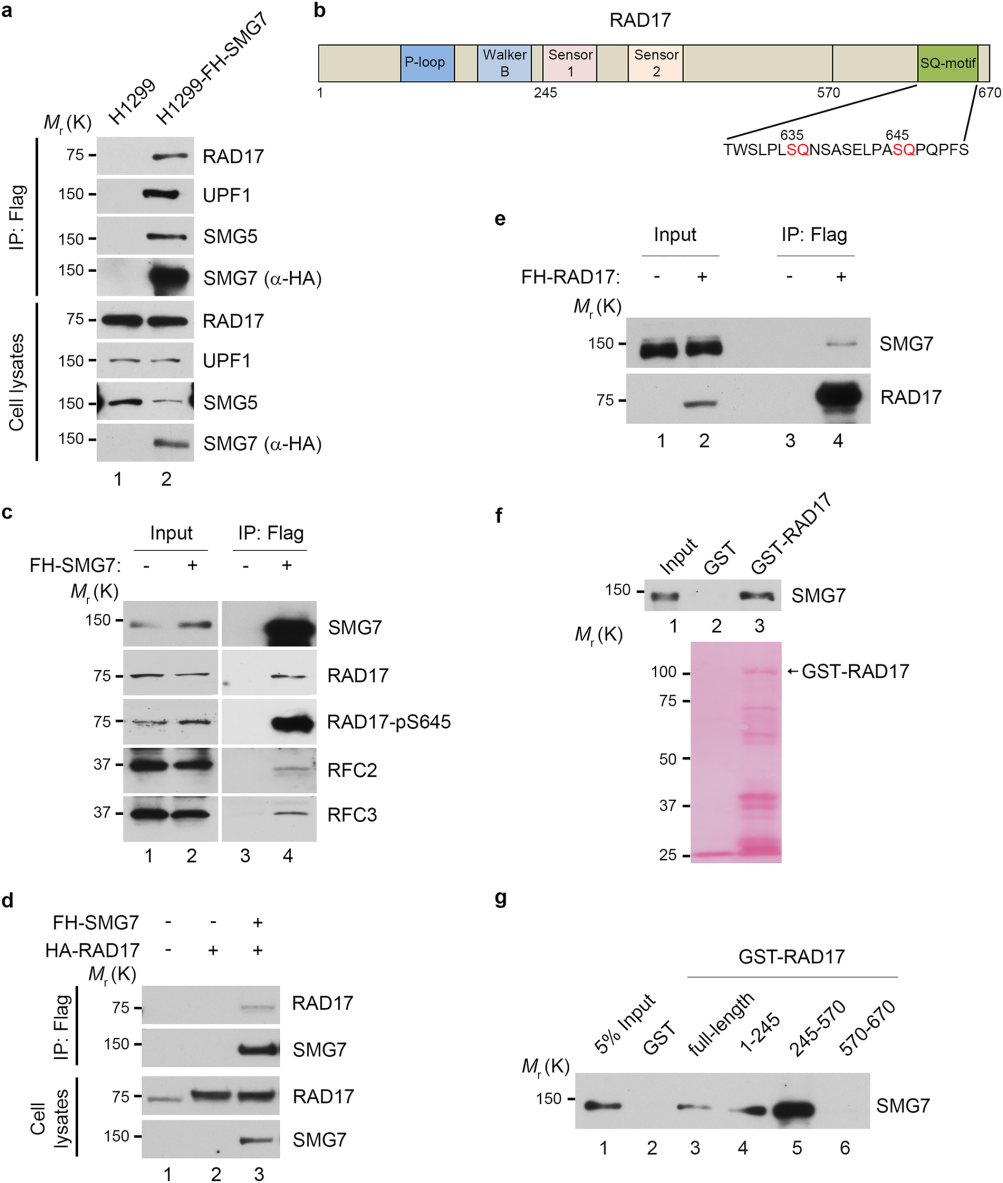
Identification of RAD17 as an SMG7-binding protein. (**a**) Verification of SMG7-binding proteins identified from complex purification. The total cell lysates and α-Flag/HA immunoprecipitated materials from H1299 and H1299-FH-SMG7 cells were assayed by western blot analysis using α-RAD17, α-UPF1, α-SMG5, and α-HA (SMG7) antibodies. (**b**) Schematic presentation of the RAD17 domain structure containing P-loop, Walker B, Sensor 1, Sensor 2 and C-terminal highlighted SQ motifs. (**c**) As in (**a**), the total cell lysates and α-Flag immunoprecipitates from H1299 and H1299-FH-SMG7 cells were assayed by western blot analysis using α-RAD17, α-RAD17-pS645, α-SMG7, α-RFC2 and α-RFC3 antibodies. (**d**) H1299 cells were transfected with SMG7- and RAD17-expressing plasmid DNA, and the cell extracts and α-Flag immunoprecipitated materials were analyzed by western blot with α-RAD17 and α-HA (SMG7) antibodies. (**e**) The total cell extracts and α-Flag immunoprecipitated materials from H1299 cells transfected with empty vector or FH-RAD17-expression plasmid DNA were examined by western blot analysis using α-SMG7 and α-RAD17 antibodies. (**f**–**g**) SMG7 direct binding to RAD17 in vitro. GST, full-length GST-RAD17 (**f**), or truncated GST-RAD17 fragment (**g**) fusion proteins were used in pulldown assays with purified FH-SMG7 proteins. FH-SMG7 was detected by western blot analysis using α-SMG7 antibody, and GST proteins visualized by Ponceau S staining in (**f**) and Supplementary Fig. 2d.

SMG7 contains an N-terminal 14-3-3-like domain and a C-terminal low complex region (LCR), both of which are implicated in mediating protein–protein interactions^35,45^. To clarify the molecular basis of CHK1 activation by SMG7, we took a proteomic approach to search for SMG7-interacting proteins that potentially regulate the ATR-CHK1 pathway. Thus, we utilized the *p53*-null human lung carcinoma H1299 cells, which have been widely used to study DNA damage and generate stable lines, to express SMG7 containing N-terminal Flag and HA (hemagglutinin) tags (FH-SMG7) (Supplementary Fig. 2a). Through tandem affinity purification using agarose beads conjugated with α-Flag and α-HA antibodies, followed by mass spectrometric analysis, we identified from the immunoprecipitates RAD17 in addition to several known SMG7-binding proteins including UPF1 and SMG5 (Supplementary Fig. 2b,c)^46^. By western blot analysis, we verified the presence of RAD17 in the SMG7-specific protein complex (Fig. 2a), indicating that RAD17 is a previously unknown SMG7-interacting protein. RAD17 contains several well-defined domains in its N-terminal region that are critical for chromatin binding and 9-1-1 loading, and two SQ motifs (S635 and S645) at its C-terminal domain (Fig. 2b)^47^. It is worth noting that SMG7 also interacts with RFC proteins, possibly via RAD17, and exhibits strong binding activity towards phosphorylated RAD17 (Fig. 2c), suggesting that SMG7 may regulate the RAD17-RFC clamp loader.

To assess the interaction of SMG7 with RAD17 further, we transiently expressed SMG7 and RAD17 in H1299 cells. As shown in Fig. 2d, RAD17 was only detected in the α-Flag immunoprecipitates from cells expressing FH-SMG7 and HA-Rad17 but not from cells expressing HA-RAD17 alone (lane 3 vs. lane 2), indicating that RAD17 was co-immunoprecipitated with SMG7. Notably, when transiently expressed, RAD17 was able to pull down endogenous SMG7 in the co-immunoprecipitation assays (Fig. 2e), suggesting a strong and stable interaction between RAD17 and SMG7 in cells. To examine whether SMG7 physically interacts with RAD17, we performed in vitro binding assays using purified recombinant proteins. As shown in Fig. 2f, SMG7 was pulled down by immobilized GST-RAD17 but not GST proteins, indicating that SMG7 binds RAD17 directly. Moreover, using various GST-RAD17 fragments, we determined that SMG7 could interact with multiple regions of RAD17 including the N-terminal 1–245 aa fragment containing the P-loop and Walker-B domain (Fig. 2g, lane 4 and Supplementary Fig. 2d) and the middle fragment containing the sensors-1 and 2 (lane 5). Thus, these data demonstrate that RAD17 is a bona fide SMG7-binding protein.

### ATR phosphorylation of RAD17 on S635 mediates its interaction with SMG7

**Figure 3:**
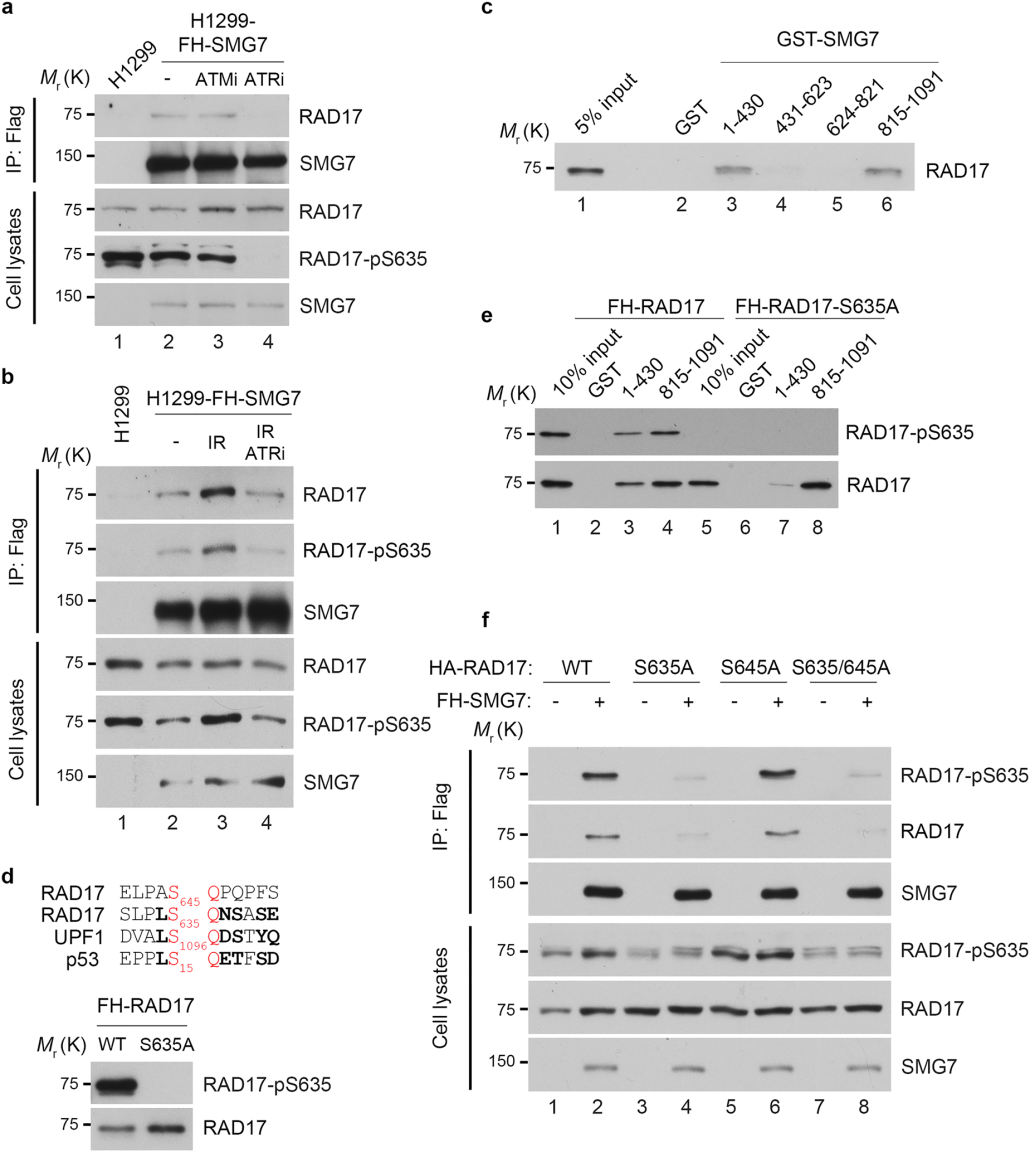
ATR-dependent RAD17 phosphorylation at S635 enhances SMG7 interaction. (**a**) The FH-SMG7-H1299 cells were treated for 4 h with 10 µM ATM (KU-55933) or ATR (VE-822) inhibitor. The total cell lysates and α-Flag immunoprecipitates were analyzed by western blot using α-SMG7, α-RAD17 and α-RAD17-pS635 antibodies. Relative levels of immunoprecipitated RAD17 are quantitated in Fig. S3a. (**b**) Cells treated with or without ATR inhibitor (VE-822, 10 µM for 4 h) were exposed to 10 Gy IR and harvested 1 h later. The total cell lysates and α-Flag immunoprecipitates were analyzed by western blot using α-SMG7, α-RAD17 and α-RAD17-pS635 antibodies as in (**a**). Relative levels of immunoprecipitated RAD17 and RAD17-pS635 are quantitated in Fig. S3b. (**c**) GST and GST-SMG7 fragment fusion proteins were incubated with lysates from 293FT cells transiently expressing HA-RAD17, and RAD17 pulled down by the GST proteins was analyzed by western blot with α-RAD17 antibody. (**d**) Alignment of the amino acid sequences adjacent to the serine residues within the SQ motif of RAD17, UPF1 and p53 (top). The FH-RAD17 and FH-RAD17-S635A proteins purified from transfected 293FT cells were analyzed by western blot using α-RAD17-pS635 and α-RAD17 antibodies (bottom). (**e**) GST or GST-SMG7 fragments were incubated with purified FH-RAD17 or FH-RAD17-S635A protein from (**d**) in GST pull-down assays. RAD17 and RAD17-pS635 were detected by western blotting with the antibodies indicated. Relative levels of RAD17 binding and RAD17-pS635 binding are quantitated in Fig. S3f. (**f**) H1299 cells were co-transfected with plasmids expressing FH-SMG7 and HA-RAD17 WT, S635A, S645A or S635/645A, and the total cell extracts and α-Flag immunoprecipitated materials were examined by western blot analysis using α-SMG7, α-RAD17 and α-RAD17-pS635 antibodies. Relative levels of RAD17 and RAD17-pS635 binding to FH-SMG7 are quantitated in Fig. S3g.

To investigate how SMG7 activates the ATR-CHK1 axis through RAD17, several lines of evidence prompted us to look into whether phosphorylation of RAD17 regulates its interaction with SMG7. These include: (1) RAD17 is phosphorylated at two SQ sites S635 and S645^27,28^; (2) these modifications are enhanced in response to genotoxic stress^27^; (3) SMG7 contains a 14-3-3 protein domain that binds phosphorylated serine; and (4) SMG7 exhibits strong binding activity towards phosphorylated RAD17 (Fig. 2c). As reported in early studies, ATM and ATR can both phosphorylate RAD17^27^; thus we tested whether ATM or ATR is required for the interaction of RAD17 with SMG7. To this end, we treated H1299 cells stably expressing FH-SMG7 with the kinase inhibitors KU-55933 (ATM) or VE-822 (ATR)^48,49^, and found that inhibition of ATR abolished phosphorylation of RAD17 on S635 and its interaction with SMG7 whereas the ATM inhibitor had no detectable effect (Fig. 3a, lane 4 vs. lane 3 and Supplementary Fig. 3a). Moreover, IR enhanced phosphorylation of RAD17 on S635 and its interaction with SMG7, which was completely abrogated by treatment with the ATR inhibitor VE-822 (Fig. 3b, lane 4 vs. lane 3 and Supplementary Fig. 3b). These data indicate that ATR is the major kinase for Rad17 S635 phosphorylation and required for the SMG7-RAD17 interaction.

Based on the strong correlation between RAD17 phosphorylation and its interaction with SMG7, we reasoned that the phosphorylated serine in the SQ motif possibly serves as a binding site for the 14-3-3 domain of SMG7. To test this hypothesis, we first mapped the RAD17-binding region of SMG7 and found that RAD17 indeed binds the N-terminal fragment containing the 14-3-3 domain as well as the C-terminal LCR (Fig. 3c, lane 3 and lane 6 and Supplementary Fig. 3). Sequence analysis of the two SQ motifs of RAD17 shows that the amino acids adjacent to S635 but not S645 are highly conserved with those of two other known SMG7-binding proteins UPF1 and p53 (Fig. 3d), which is consistent with the finding that loss of S645 phosphorylation had no effect on SMG7 binding (Supplementary Fig. 3d). To examine whether RAD17 S635 contributes to SMG7 binding, we carried out in vitro pulldown assays using purified wild type RAD17 proteins, which are readily phosphorylated on S635 when expressed in cells, and mutant RAD17 with S635 replaced with alanine (Fig. 3d). Interestingly, we found that loss of S635 phosphorylation markedly reduced RAD17 binding to the SMG7 14-3-3 domain (Fig. 3e, lane 3 vs. lane 7 and Supplementary Fig. 3e,f), but only had mild effects on its binding to the LCR region (Fig. 3e. lane 4 vs. lane 8 and Supplementary Fig. 3f). Moreover, when exogenously expressed in cells, wild type RAD17 bound SMG7 much more strongly than the RAD17 S635A mutant (Fig. 3f, lane 2 vs. lane 4 and Supplementary Fig. 3g), and the reduced binding was not further exacerbated by the S645A mutation (lane 4 vs. lane 8). It is worth noting that the S635-phosphorylated RAD17 detected in the immunoprecipitated materials from cells expressing S635A or S635A/S645A mutants is likely endogenous RAD17 (Fig. 3f, lanes 4 and 8), as transiently expressed SMG7 was able to pull down endogenous S635-phosphorylated RAD17 (Supplementary Fig. 3h). Taken together, these data indicate that ATR phosphorylation of RAD17 on S635 is critical for its interaction with SMG7 under both normal and genotoxic stress conditions.

### SMG7 promotes chromatin recruitment of RAD9 upon DNA damage

**Figure 4:**
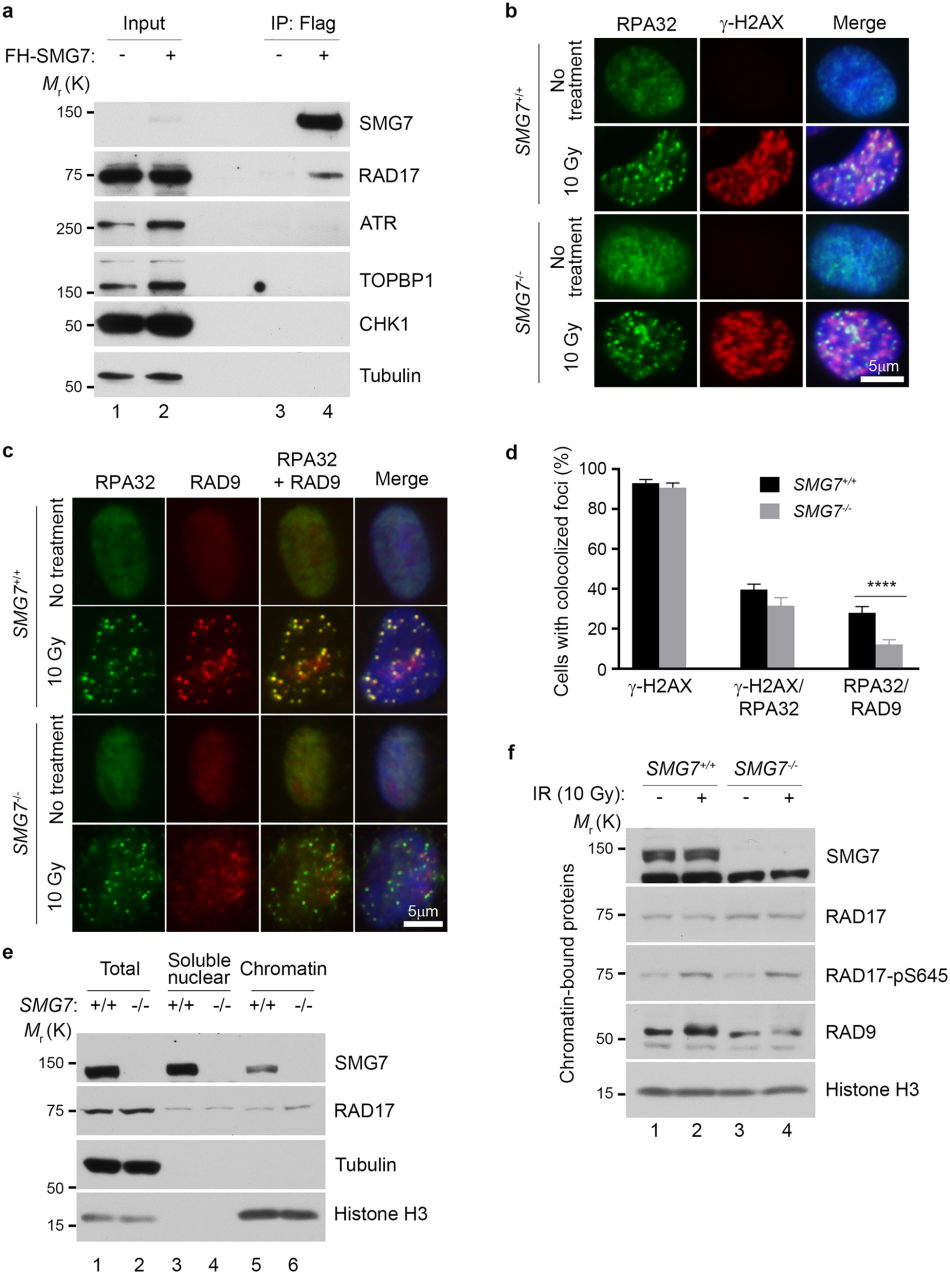
SMG7 promotes chromatin recruitment of RAD9 upon DNA damage. (**a**) Cell extracts from DLD1 *SMG7*^*−/−*^ cells expressing FH-SMG7 and the α-Flag immunoprecipitates were examined by western blot analysis using α-SMG7, α-RAD17, α-ATR, α-TOPBP1, α-CHK1 and α-Tubulin antibodies. (**b**,**c**) Wild type and *SMG7*^*−/−*^ cells were treated with ionizing radiation (10 Gy, 1 h), pre-extracted, and immunostained in (**b**) with α-RPA32 (green) and α-γH2AX (red) antibodies and in (**c**) with α-RPA32 (green) and α-RAD9 (sc-74464, red). Nuclei were stained with DAPI. Representative images from immunofluorescence microscopy are shown. Wider fields of view at are shown in Fig. S4b-c. (**d**) Quantification of yH2AX-, yH2AX/RPA- and RPA/RAD9- foci-positive cells from experiments represented in (**b**,**c**). Data are presented as Mean ± SEM (n = 3 independent experiments containing pooled data) and were analyzed by one-way ANOVA with Tukey post-test. **** indicates *P* < 0.001. (**e**) Wild type and *SMG7*^*−/−*^ DLD1 cells were subjected to fractionation, and the total cells extracts, soluble nuclear and chromatin fractions were analyzed by western blot using α-SMG7 and α-RAD17 antibodies. α-alpha-Tubulin and α-Histone H3 antibodies were used as cytoplasmic and chromatin markers, respectively. Relative levels of chromatin-bound SMG7 and RAD17 are quantitated in Fig. S4d. (**f**) As in (**e**), the chromatin-bound fractions from the control and irradiated cells were isolated and examined by western blot analysis using α-SMG7, α-RAD17, α-RAD17-pS645, α-RAD9 (A300-890A) and α-H3 antibodies. Relative levels of chromatin-bound RAD9 are quantitated in Fig. S4f.

So far, our data suggest that SMG7 activates the ATR-CHK1 axis upon DNA damage through interactions with RAD17, and this protein binding activity appears to be very specific for RAD17, as we did not observe any interactions between SMG7 and ATR, CHK1, or TOPBP1 in HCT116 and DLD1 cells (Fig. 4a, and Supplementary Fig. 4a). A key function of RAD17-RFC in the activation of ATR-CHK1 is to load the 9-1-1 clamp^16,21^, and given that SMG7 interacts with RFC proteins as well, we examined whether SMG7 regulates the recruitment or retention of RAD9 to the DNA damage site. To this end, we performed immunofluorescence staining of cells following DNA damage, and found that IR induced similar levels of DSBs (~ 90% γ-H2AX positive cells) in wild type and *SMG7*^*−/−*^ cells (Fig. 4b and Supplementary Fig. 4b). In addition, compared with wild-type cells, only a very mild reduction in the number of *SMG7*^*−/−*^ cells with γ-H2AX/RPA32 foci was observed, which suggests that loss of SMG7 may have, if any, a slight effect on the formation of ssDNA following DSB resection. These data are largely consistent with our observation that loss of SMG7 does not affect activation of ATM (Fig. 1a,b), which controls DSB resection^50,51^. As formation of RPA-ssDNA is the starting point of ATR activation, the presence of RPA32 foci indicates that the initial molecular signal for ATR-CHK1 activation is present in wild type and *SMG7*^*−/−*^ cells. Interestingly, although IR strongly induced RAD9 foci co-localized with the RPA32 foci in wild type cells, the RAD9/RAP32-colocolized foci were much less pronounced in the *SMG7*^*−/−*^ cells (Fig. 4c and Supplementary Fig. 4c) and the *SMG7*^*−/−*^ cells also had significantly less RAD9/RAP32-colocolized foci fractions than wild type cells (Fig. 4d and Supplementary Fig. 4c), suggesting that SMG7 is important for the recruitment or retention of the RAD9 complex to the DNA damage site.

To corroborate these findings, we carried out chromatin fractionation assays to examine RAD9 recruitment upon DNA damage^52^. Notably, we found that in unstressed cells, a small fraction of SMG7 was associated with chromatin, and loss of SMG7 had no effect on the levels of the chromatin-bound RAD17 (Fig. 4e, lane 5 vs. lane 6 and Supplementary Fig. 4d,e). Interestingly, the chromatin-bound fraction of RAD9 was substantially reduced in the *SMG7*^*−/−*^ cells compared with wild type cells following IR (Fig. 4f, lane 2 vs. lane 4 and Supplementary Fig. 4f), which is consistent with the results from immunofluorescence staining. Thus, these data show that SMG7 plays an important role in recruitment or retention of the RAD9 complex to the DNA damage site and reveals a novel mechanism for SMG7 activation of the ATR-CHK1 axis.

### SMG7 regulates CHK1 activation and fork progression following replication stress

**Figure 5:**
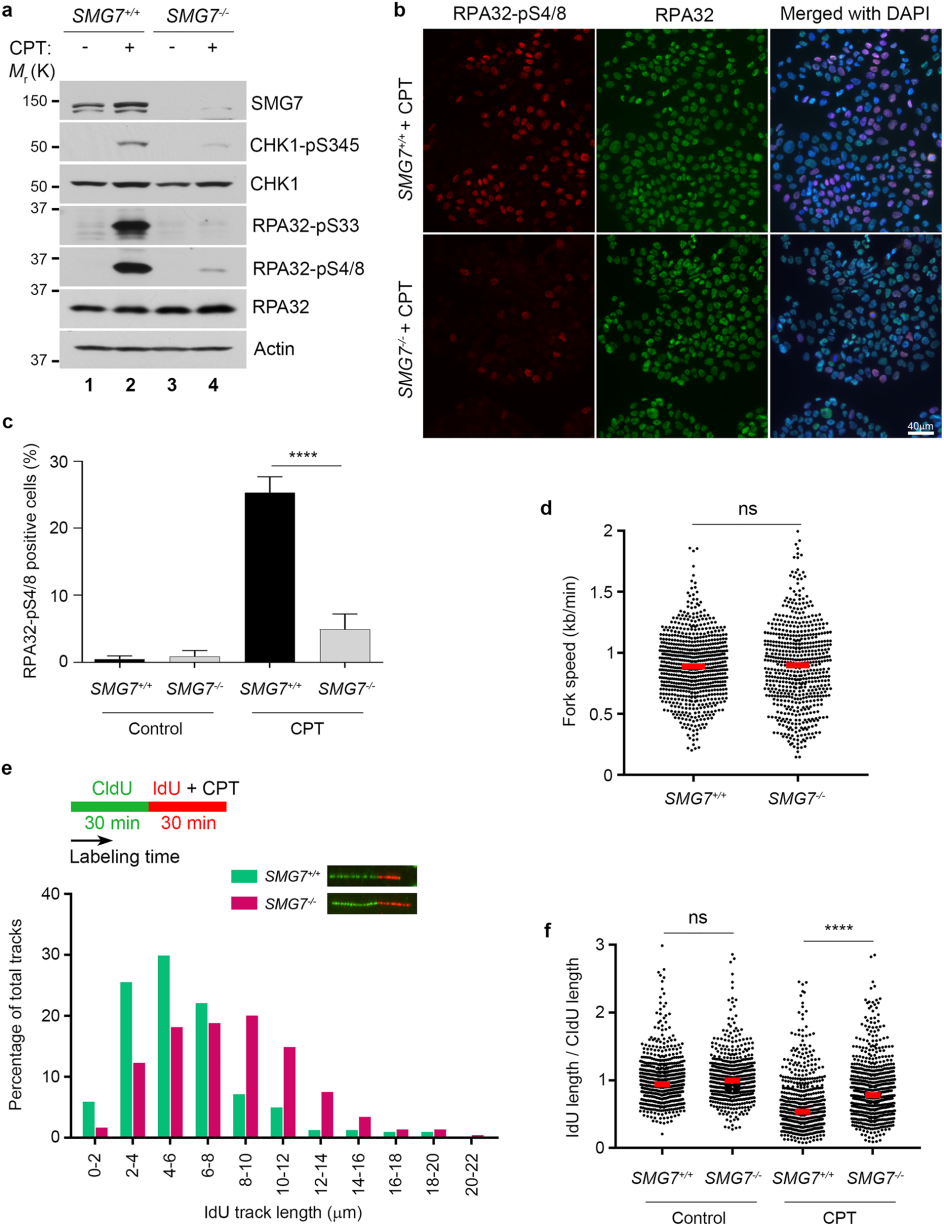
SMG7 regulates ATR signaling and CHK1 activation and fork progression following replication stress. (**a**–**c**) Wild type and *SMG7*^*−/−*^ cells were treated with 1 µM CPT for 1 h. (**a**) Total cell extracts were examined by western blot analysis using α-SMG7, α-CHK1, α-CHK1-pS345, α-RPA32-pS33, α-RPA32-pS4/8, α-RPA32, and α-Actin antibodies. Relative levels of CHK1-pS345, RPA-pS33, and RPA-pS4/8 in CPT-treated cells are quantitated in Fig. S5c. (**b**) Cells were immunostained with α-RPA32-pS4/8 (red) and α-RPA32 (green) antibodies. Representative images from immunofluorescence microscopy are shown. (**c**) Quantification of S4/8-positive cells from (**b**) using MetaMorph software. Data are presented as Mean ± SEM (n = 3 independent experiments containing pooled data) and analyzed by one-way Anova with Tukey post-test; **** indicates *P* < 0.001. Graphs were generated using GraphPad Prism. (**d**–**f**) DNA fiber analysis of untreated or CPT-treated wild type and *SMG7*^*−/−*^ cells as in (**a**). Cells were first labeled with 25 μM CldU for 30 min and then by 250 μM IdU for 30 min in the absence or presence of CPT. DNA fibers were then spread, stained with α-BrdU antibodies (B44 and BU1/75) and visualized using fluorescence microscopy. More than 500 CIdU-/IdU-labeled tracks from two independent experiments were measured using ImageJ. (**d**) Quantification of replication fork speed using the lengths of CldU- and IdU-labeled DNA fiber. Fork speed was estimated using 1 μm = 2.6 kbp as described in Methods. Student’s t-test was performed to determine the statistical difference; n.s. not significant. (**e**) Scheme for the CIdU/IdU labeling and CPT treatment (Top). Representative tracks were shown (Middle). Distribution of the IdU-labeled tracks from the CPT-treated cells (Bottom). (**f**) Quantification of length ratios of the IdU-labeled tracks to the CldU-labeled tracks per replication fork. Red bars represent the means of each population. Data were analyzed by one-way Anova with Tukey post-test; **** indicates *P* < 0.001; n.s. not significant, *P* > 0.05.

The ATR-CHK1 axis is a key regulator of replication stress, upon which activated CHK1 halts DNA replication in the S phase of the cell cycle by inhibiting replication initiation and progression of the replication fork^41,42,53^. Thus, our observation that loss of SMG7 impaired activation of CHK1 following IR (Fig. 1) suggests that SMG7 has an important function in the regulation of the cellular response to replication stress induced by DNA lesions. To assess the role of SMG7 in S phase, we utilized camptothecin (CPT), a topoisomerase I inhibitor that specifically induces DSBs on replicating DNA^54^. As expected, treatment with CPT induced γ-H2AX and RPA32 foci similarly in both wild type and *SMG7*^*−/−*^ cells (Supplementary Fig. 5a,b), suggesting that SMG7 does not affect formation of RPA-bound ssDNA. Interestingly, loss of SMG7 significantly reduced CPT-induced phosphorylation of CHK1 on S345 (Fig. 5a, lane 2 vs. lane 4, Supplementary Fig. 5c). Moreover, phosphorylation of RPA32 on S33 and S4/8 following treatment with CPT was nearly abolished in the absence of SMG7 (Fig. 5a, lane 2 vs. lane 4, Fig. 5b,c, and Supplementary Fig. 5c), indicating that SMG7 is critical for activation of the ATR/CHK1 signaling upon replication stress.

To examine how SMG7 regulates replication stress, we performed DNA fiber analysis, a well-established double-labeling approach that uses the BrdU derivatives CldU and IdU to directly assess the replication fork^55^. This dual labeling strategy makes it possible to (1) identify active replication forks during the labeling times, which proceed bi-directionally from their origins, and (2) determine the effect of replication stress on fork progression by measuring the IdU-labeled DNA tracks. To this end, cells were pulse-labeled with CldU and IdU (with or without CPT), followed by DNA spreading, immunostaining and microscopic analysis for the presence and length of the distinct CIdU and IdU tracks. Under normal conditions, wild type and *SMG7*^*−/−*^ cells exhibited similar symmetric CIdU/IdU-labeled tracks (Supplementary Fig. 5d) and showed no significant difference in fork speed (Fig. 5d). Interestingly, in the presence of CPT, wild type cells had much shorter IdU-labeled tracks compared with the *SMG7*^*−/−*^ cells (Fig. 5e, distribution within 2–8 µm for wild type and 4–12 µm for the *SMG7*^*−/−*^ cells, respectively), which is consistent with previous studies showing that activation of CHK1 inhibits fork progression^56^. Moreover, by analyzing hundreds of active replication forks individually, we found that treatment with CPT resulted in a significant reduction in the ratio between IdU and CIdU-labeled tracks, which was reversed by inhibition of CHK1 or loss of SMG7 (Supplementary Fig. 5e,f). Thus, these data indicate that by promoting ATR signaling and CHK1 activation, SMG7 has a important role in the control of fork progression in response to replication stress.

### SMG7 regulates CHK1-S345 phosphorylation and modulates cell cycle progression during recovery from replication stress

The crucial function of CHK1 in the replication stress response is manifested not only in fork regulation but also during recovery of cells after removal of stress conditions^56–61^. During the process of recovery, the ATR-mediated checkpoint signaling is attenuated in a regulated manner, which ensures coordinated DNA replication and cell cycle progression after replication stress is resolved^62^. Not surprisingly, deficiency in CHK1 itself or components required for activation of the ATR-CHK1 axis impairs normal cell cycle progression or cell survival after cells are relieved from a short-term replication stress of reversible nature, such as a few hours of treatment with hydroxyurea (HU), a ribonucleotide reductase inhibitor that induces replication stress by depleting cellular deoxyribonucleotides. For example, cells depleted of RAD17 or expressing phosphorylation-defective S635/645A mutant RAD17 are unable to maintain controlled CHK1 activation and lose cell viability during recovery from HU treatment^29^. Thus, our finding that SMG7 promotes CHK1 activation through robust binding to S635-phosphorylated RAD17 prompted us to examine whether SMG7 is involved in facilitation of replication stress recovery. As expected, HU treatment strongly induced CHK1 activation in a dose- and time-dependent manner, which was markedly reduced in the absence of SMG7 (Supplementary Fig. 6a,b). When we assessed ATR phosphorylation of CHK1 shortly after HU removal, we found that levels of S345-phosphorylated CHK1 decreased over time in wild type cells, and loss of SMG7 further accelerated this downregulation (Supplementary Fig. 6c). Consistent with previous studies^29^, these data suggest that SMG7 plays an important role in maintaining CHK1 S345 phosphorylation in the early stages following removal of replication stress.

**Figure 6:**
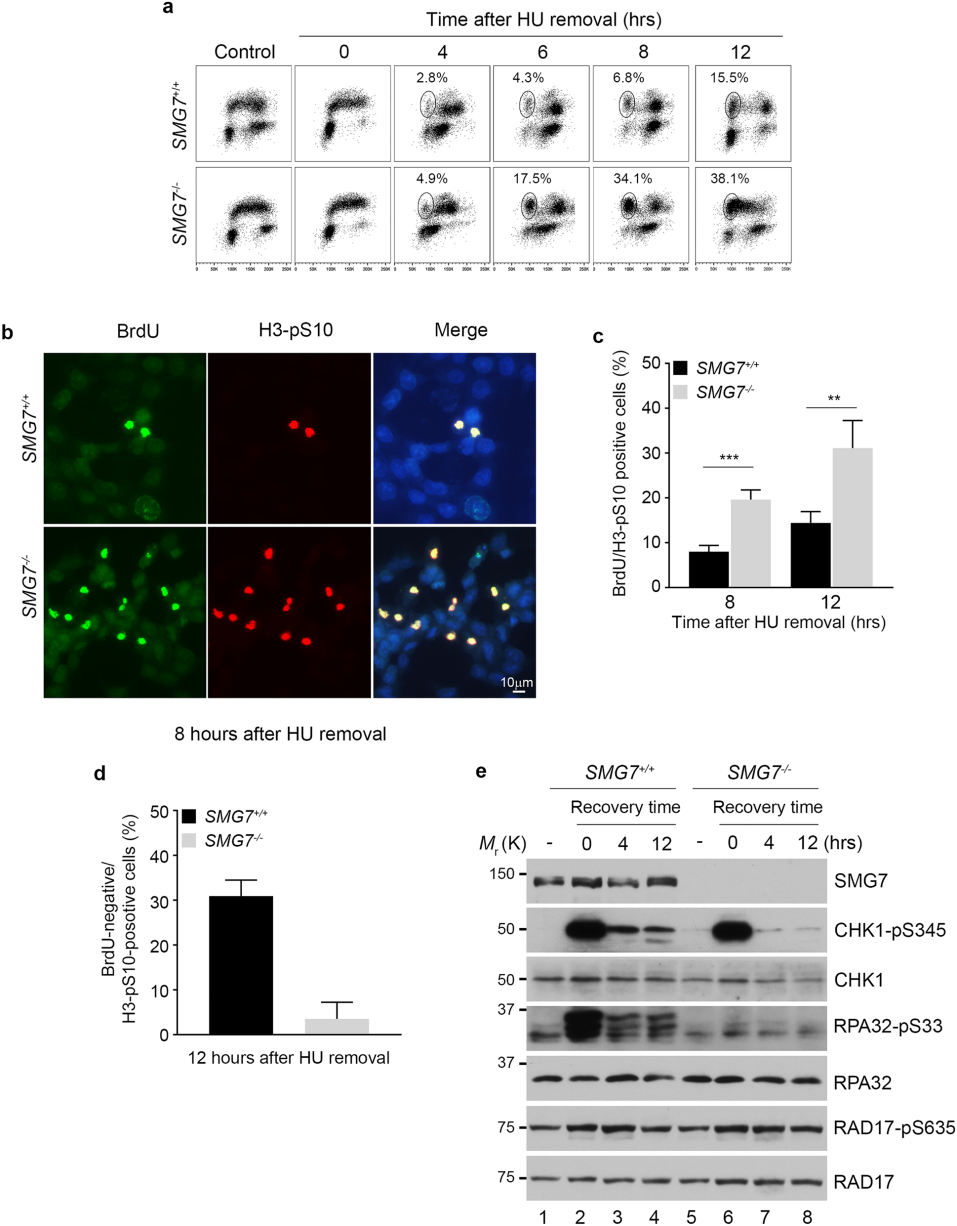
SMG7 maintains normal attenuation of the ATR-CHK1 axis during recovery from replication stress. (**a**) Wild type and *SMG7*^*−/−*^ HCT116 cells were pulsed with 25 μm BrdU followed by treatment with 5 mM HU for 6 h. After HU treatment, cells were released into fresh normal media, and harvested at the indicated time points. Cells were then fixed, stained with α-BrdU antibody (y-axis) and 7-AAD (x-axis) and analyzed by flow cytometry. The BrdU-positive fractions containing G_1_-DNA content from total populations are circled (4, 6 and 8 h after HU removal). (**b**–**d**) Cells treated as in (**a**) were released into fresh media containing 1 μg/mL nocodazole for different hours. Cells were then fixed and stained with α-BrdU (BU1/75) (green) and α-Histone H3-pS10 (red) antibodies, and imaged using a fluorescence microscope. Representative images of cells 8 h after HU removal are shown in (**b**). Cells were counted using ImageJ, and the percentage of BrdU/H3-pS10 double positive (**c**) cells were quantified. Data are presented as Mean ± SD (n = 3) and analyzed by one-way ANOVA (****P < 0.0001). (**d**) BrdU-negative/H3-pS10-positive cells were quantified. Data are presented as Mean ± SEM (n = 3) and analyzed by Student’s t-test; *P* < 0.01. (**e**) Total cell extracts from wild type and *SMG7*^*−/−*^ cells treated as in (**b**–**d**) were examined by western blot analysis using α-SMG7, α-CHK1-pS345, α-CHK1, α-RPA32-pS33, α-RPA32, α-RAD17 and α-RAD17-pS635 antibodies.

Next, we examined cell cycle profile, and found that when the whole cell population was analyzed in a course of 12-h recovery, the *SMG7*^*−/−*^ cells showed deficient cell cycle progression (Supplementary Fig. 6d). To assess the S-phase population specifically, we pulse-labeled cells with BrdU, and then treated them with HU, which arrested BrdU-positive cells throughout the S phase and BrdU-negative cells at G1 (Fig. 6a, control vs. 0 h). Until 4 h after HU removal, we observed little difference in the S-phase progression of the BrdU-positive cells. The gradual increase (from 4 to 8 h) in the wild type BrdU-positive population with 2 N DNA content, which results from completion of mitosis, indicates a regulated and orderly entry into mitosis from G_2_ after release from HU. Interestingly, the *SMG7*^*−/−*^ BrdU-positive cells completed the G_2_-M transition in an accelerated fashion, and subsequently accumulated at the 2 N position (Fig. 6a; *SMG7*^*−/−*^ 34.1% vs. wild type 6.8% by 8 h). The accelerated transition of the *SMG7*^*−/−*^ BrdU-positive cells was clearly illustrated when the BrdU-positive population was selected and analyzed by flow cytometry (Supplementary Fig. 6e). To assess the role of SMG7 in the G_2_-M progression directly during the 12-h course of recovery, we treated cells with the mitosis-arresting agent nocodazole upon HU removal and assayed the presence of the mitotic marker S10-phosphorylated histone 3 (H3-pS10). Notably, the BrdU-positive population of the *SMG7*^*−/−*^ cells had a significantly higher H3-pS10-positive fraction compared with that of wild type cells (Fig. 6b,c), suggesting that SMG7 is indeed critical for the G_2_-M transition during recovery from replication stress. It is worth noting that the BrdU-negative population of wild type cells showed a higher H3-pS10-positive fraction than that of the *SMG7*^*−/−*^ cells (Fig. 6d), and this is likely because wild type cells appeared to progress from G_1_ through S phase more quickly (Fig. 6a).

To determine the underlying mechanism by which SMG7 regulates the G_2_-M transition, we examined the status of CHK1 phosphorylation during recovery, as previous studies have shown that the mitotic entry from G_2_ is controlled by CHK1^57,59,60^. While treatment with a higher dose of HU (5 mM) for a prolonged period of time (6 h) induced similar CHK1 S345 phosphorylation regardless of SMG7 status, importantly, wild type cells retained markedly higher levels of S345-phosphorylated CHK1 than *SMG7*^*−/−*^ cells after removal of HU (Fig. 6e, lanes 3–4 vs. 7–8). The further reduced CHK1 S345 phosphorylation in the absence of SMG7 during recovery is consistent with the observation that *SMG7*^*−/−*^ cells showed an accelerated G_2_-M transition. It is also interesting to note that loss of SMG7 abolished HU-induced phosphorylation of RPA32 on S33 but had no effect on RAD17 phosphorylation on S635 before or after HU removal. Thus, these data indicate that SMG7 plays an important role in regulating CHK1 S635 phosphorylation and modulating cell cycle progression during recovery from replication stress.

## Discussion

Here, we report a previously unknown function of SMG7 in regulation of the ATR-CHK1 pathway in response to DNA damage and replication stress. For the first time, we have identified SMG7 as a binding partner to the RAD9-RAD1-HUS1 clamp loader protein RAD17 and shown that SMG7 promotes the recruitment of RAD9 to RPA-coated ssDNA in the activation of CHK1. The interaction of SMG7 with RAD17 is highly dynamic and tightly controlled by the ATR-dependent phosphorylation of RAD17 on S635, a conserved SQ site that is required for RAD17 checkpoint function. Importantly, loss of SMG7 impairs ATR signaling and CHK1 activation upon genotoxic stress and disables intra-S checkpoint function in control of replication fork progression. Furthermore, SMG7 has an important role in maintaining CHK1 S345 phosphorylation and cell cycle progression. In summary, our present studies identify SMG7 as an indispensable signaling component in activation of the ATR-CHK1 pathway through RAD17 binding and RAD9 recruitment, and suggests that by engaging phospho-SQ-mediated signaling through its 14-3-3 domain, SMG7 has a more general and direct role in regulation of the genotoxic stress response.

Activation of the ATR-CHK1 axis upon DNA damage is a complex process, which necessitates recruitment of the 9-1-1 complex to the damage site by RAD17-RFC^2,5,8^. Given that loss of SMG7 markedly impairs the formation of RAD9 foci in regions of RPA-bound ssDNA, our data provide an important insight into how SMG7 engages in the ATR-CHK1 signaling pathway (Fig. 4c). Although the precise mechanism by which SMG7 promotes RAD9 recruitment/retention remains to be fully elucidated, SMG7 binding to RAD17 and other RFC subunits likely has a direct impact on 9-1-1 association and/or recruitment (Fig. 2). For example, studies have shown that the N-terminal P-loop and Walker-B domains of RAD17 are critical for 9-1-1 binding and recruitment^47,63^; thus the direct binding of SMG7 to the RAD17 N-terminal region may facilitate 9-1-1 recruitment or stabilize the RAD17 interaction with 9-1-1. Additionally, SMG7 may promote the recruitment/retention of the RAD9 complex through interaction with the S635-phosphorylated RAD17. While early reports suggest that ATR phosphorylation of RAD17 is not required for initial RAD9 loading, recent studies show that phosphorylation of the RAD17 SQ motif indeed plays an important role in maintaining DNA damage-induced RAD9 foci^22,30^. Therefore, it is likely that through interacting with S635-phosphorylated RAD17, SMG7 may stabilize RAD9 foci following its recruitment, which allows full activation of the ATR-CHK1 axis. Besides acting as a RAD9-RAD1-HUS1 clamp loader protein, RAD17 also functions in CHK1 recruitment by CLASPIN, which is critical for ATR phosphorylation and activation of CHK1^29,64^. Intriguingly, SMG7 and CLASPIN both engage with RAD17 in a phosphorylation-dependent manner via interactions with phosphorylated RAD17, but it is not known whether S635, S645 or both are required for the RAD17-CLASPIN interaction. While we show that loss of SMG7 reduces RAD9 recruitment/retention (Fig. 4), our data does not rule out the possibility that SMG7 may be involved in CLASPIN regulation of CHK1. It is possible that SMG7 may compete with CLASPIN in RAD17 binding; however, given that both SMG7 and CLASPIN positively regulate CHK1, it is intriguing how these two RAD17 binding proteins may affect each other with respect to different aspects of CHK1 activation. For example, CLASPIN is critically important for the attenuation of the ATR-CHK1 signaling during recovery^57,59^ and it remains to be elucidated whether and how SMG7/phosphorylated RAD17 are involved in this regulation.

RAD17 is an important checkpoint regulator, and its phosphorylation by ATR and ATM appears to be a pivotal signaling step in shaping the response of a cell to genotoxic stress^27,29,31^. As reported here and previously, ATR is the major kinase that phosphorylates RAD17 on the SQ sites S635/645, and loss of these modifications impairs the RAD17 checkpoint function^27,29^. Moreover, a recent study has shown that RAD17 activates ATM in response to DSB through binding to Nijmegen breakage syndrome (NBS1) of the MRE11-RAD50-NBS1 (MRN) complex, which involves the phosphorylation of RAD17 on T622 by ATM^31^. Thus, our finding that SMG7 interacts with RAD17 in an ATR-dependent but ATM-independent manner reveals phosphorylation-mediated SMG7 binding to RAD17 as a potential underlying mechanism by which SMG7 exerts its function in response to genotoxic stress (Fig. 3a,b). Indeed, our data show that loss of SMG7 abrogates CHK1 activation but has no effect on the ATM-CHK2 pathway, and support a model in which through binding to S635-phosphorylated RAD17 in an ATR-dependent fashion, SMG7 acts on ATR-CHK1 signaling following DNA damage (Figs. 1a–c and 3e,f). Importantly, consistent with its role in activation of the ATR-CHK1 axis, loss of SMG7 disables CHK1-dependent intra-S checkpoint function in control of replication fork progression in response to CPT-induced replication stress (Fig. 5). However, it appears that SMG7 is not significantly involved in regulation of CHK1 under basal conditions, as indicated by normal DNA replication and fork progression in *SMG7*^*−/−*^ cells during S phase (Figs. 1d,e and 5). Moreover, unlike *RAD17*-null cells, which rapidly lose viability following deletion of RAD17^65^, *SMG7*^*−/−*^ cells are able to grow and proliferate normally, consistent with the phenotype observed in cells expressing the phosphorylation-defective S635/645A RAD17 mutant^29^. Thus, these data support the notion that SMG7 functions in the ATR-CHK1 pathway mainly, if not exclusively, through interaction with the S635-phosphorylated RAD17.

In addition to being indispensable for CHK1 activation upon DNA damage, our data further show that SMG7 may have an important role in regulating CHK1 and cell cycle progression during recovery from replication stress. Unlike wild type cells, *SMG7*^*−/−*^ cells fails to retain CHK1 S345 phosphorylation shortly after release from replication stress, consistent with a previous study showing that loss of RAD17 phosphorylation on S635/645A leads to a defect in maintaining Chk1 activation after HU withdrawal^29^. Furthermore, *SMG7*^*−/−*^ cells exhibit an accelerated transition from G2 to M after release from prolonged treatment (6 h) with a higher dose of HU (5 mM), which is supported by data that *SMG7*^*−/−*^ cells have even lower S345-phosphorylated CHK1 compared to wild-type cells during recovery (Fig. 6 and Supplementary Fig. 6). As S635/645-phosphorylated RAD17 engages both SMG7 and CLASPIN, whose degradation is critical for CHK1 inactivation during recovery from replication stress^29^, it is possible that SMG7 may regulate CHK1 by modulating CLASPIN function during recovery from replication stress. It is important to note that in these experiments HU treatment (5 mM for 6 h) induced similar CHK1 S345 phosphorylation regardless of SMG7 status, suggesting that wild-type and SMG7^*−/−*^ cells have the same levels of initial CHK1 activation following HU treatment. As our data show that loss of SMG7 does not abolish CHK1 S345 phosphorylation after HU treatment and only attenuates it in a dose- and time-dependent manner, indicating that the mechanism for CHK1 activation still exists in SMG7^*−/−*^ cells (Supplementary Fig. 6a,b). It is worth noting that the level of CHK1 phosphorylation in SMG7^*−/−*^ cells treated with 2 mM HU for 1 h is even higher than that in wild-type cells treated with 0.5 mM HU. A recent study shows that the amount of HU and the time of its treatment has very dynamic effects on the recruitment pattern of several critical proteins involved in CHK1 activation such as TOPBP1 and the 9-1-1 complex^66^. Thus, it is conceivable that under specific conditions such as prolonged treatment with a high concentration (5 mM) of HU, CHK1 S345 phosphorylation may be maximized in both wild-type and SMG7^*−/−*^ cells, which leads to similar levels of S345-phosphorylated CHK1.

In response to genotoxic stress, ATR phosphorylates a plethora of proteins, and there is enormous interest in understanding the mechanism for ATR activation towards the different substrates^3,5,8^. By examining CHK1 and RPA32, a recent study has identified two distinct ATR activation modes, which are orchestrated by RAD17 and NBS1 in phosphorylation of CHK1 and RPA32, respectively^67^. Interestingly, our present data show that loss of SMG7 abolishes ATR phosphorylation of CHK1 and RPA32 but has no effect on RAD17 (Figs. 1a–c, 4f, 5a and 6a,f), suggesting that SMG7 may regulate phosphorylation of ATR substrates differentially, and likely engages either directly or indirectly other components (e.g., NBS1) of the ATR pathway besides RAD17. Future studies aimed to elucidate the precise mechanisms of SMG7-mediated signaling will further our understanding of the role of SMG7 in the regulation of genotoxic stress response.

## Methods

### Cell culture

The human colorectal adenocarcinoma HCT116, DLD1 and their *SMG7*-null derivative cell lines were cultured in McCoy’s 5A medium (Corning, Manassas, VA, USA) supplemented with 10% fetal bovine serum (Sigma, F2442). The H1299 and 293FT cells were cultured in Dulbecco’s modified Eagle’s medium (DMEM, Corning/Cellgro) supplemented with 10% fetal bovine serum and combined antibiotics (100 I.U/ml penicillin and 100 ug/ml streptomycin).

### Generation of DLD1 *SMG7*^*−/−*^ cell line

We carried out CRISPR-Cas9-mediated gene targeting in DLD1 cells based on the protocol described in the previous study^68^. Briefly, two sgRNAs (target sequence: 5′- ATGTTCGAATAATCAGATTG-3′ and 5′-GATTATAGGTGATCAATCCC-3′) designed using the online CRISPR Design Tool (http://tools.genome-engineering.org) were cloned into the Cas9-expressing plasmid PX458 (Addgene, #48138). After transfection with the sgRNA-expressing plasmid DNA and pCin4-puro (Clontech, #6031-1), cells were treated with 3 µg/ml puromycin for 24 h and plated in 96-well in the absence of puromycin. Two weeks later, clones were expanded and screened for SMG7 targeting, which was further verified by DNA sequencing.

### Plasmids construction

pCin4-FH-SMG7, pCin4-FH-RAD17 and pCin4-HA-RAD17 plasmids were constructed by cloning the cDNA encoding SMG7 or RAD17 generated by RT-PCR from mRNA extracted from HCT116 cells into pCin4-Flag-HA or pCin4-HA vectors. pCin4-FH-RAD17-S635A, pCin4-HA-RAD17-S635A, S645A and S635A/645A plasmids were generated by site-directed mutagenesis. GST-RAD17 full-length, GST-RAD17 fragments and GST-SMG7 fragments expressing plasmids were generated by cloning of each DNA fragments into pGEX-2TL(+) vector. All the plasmids were confirmed by DNA sequence analysis.

### Antibodies and inhibitors

The following antibodies were from Bethyl: α-H2AX (A300-081A, 1:1000), α-H2AX (A303-837A, 1:2000), α-RAD9 (A300-890A, WB 1:500, IF 1:50), α-RAD17-pSer645 (A300-153A, 1:1000), α-RFC2 (A300-142A, 1:1000), α-RFC3 (A300-188A, 1:1000), α-RPA32-pS4/8 (A300-245A, 1:1000), α-RPA32-pS33 (A300-246A, 1:1000), α-RPA32 (A300-244A, 1:3000), α-SMG7 (A302-170A, 1:1000). The following antibodies were from Cell Signaling Technology: α-ATM-pS1981 (5883, 1:1000), α-ATM (2873, 1:1000), α-CHK1-pS345 (2348, 1:1000), α-CHK2-pT68 (2661, 1:1000), α-CHK2 (2662, 1:1000), α-RAD17-pS635 (13404S, 1:1000), α-RAD17 (8561, 1:1000), α-RPA32 (2208S, IF 1:1000), α-Histone H3 (3638, 1:2000), α-rabbit IgG-HRP (7074, 1:5000). The following antibodies were from Santa Cruz Technology: α-Actin-beta (sc-47778, 1:1000), α-ATR (sc-1887, 1:1000), α-CHK1 (sc-8408, 1:100), α-Histone H3 p-Ser10 (sc-8656, IF 1:200), α-RAD9 (sc-74464, IF 1:100), α-RAD17 (sc-17761, 1:500), α-Tubulin-alpha (sc-53029, 1:1000). Goat α-rat IgG-HRP (3050-05, 1:5000) and donkey α-goat IgG-HRP (6420-05, 1:5000) were from Southern Biotech. α-ATR-pT1989 (GTX128145, 1:1000), α-HA (MMS-101P, 1:1000), α-SMG5 (12694-1-P, 1:1000), α-SMG7 (LS-C353204-100, 1:1000) and Sheep α-mouse IGG-HRP (NA931, 1:5000) were from GeneTex, Bio-Rad, Covance, Proteintech, LifeSpan Biosciences and GE healthcare, respectively. Antibodies used for BrdU staining include α-BrdU clone B44 (347580, 1:200; BD Biosciences) and α-BrdU clone BU1/75 (MCA2060, 1:200 BioRad). The following inhibitors were used: ATR inhibitor (VE-822; MedKoo Biosciences) and ATM inhibitor (KU-55933; Sigma-Aldrich).

### Purification of the SMG7-associated protein complex

The H1299-FH-SMG7 stable cell lines were generated by transfection with pCin4-FH-SMG7, followed by selection with 1 mg/ml G418 for 2–3 weeks. To purify the SMG7 protein complex, freshly grown H1299-FH-SMG7 cells (~ 5 × 10^8^) were harvested in cold PBS. The cell pellets were suspended in 20 times volume of ice cold BC100 buffer (20 mM Tris–HCl pH 7.9, 100 mM NaCl, 10% glycerol, 0.2 mM EDTA and 0.5% Triton X-100) with fresh proteinase inhibitor cocktail and incubated on ice for 1 h with several times of brief vertex. After centrifugation at 15,000 rpm for 30 min at 4 °C, the supernatants were cleared by passing through 0.45 μm syringe filters and the final cell extracts were subjected to immunoprecipitation with anti-Flag antibody-conjugated M2 agarose beads (Sigma). The bound polypeptides eluted with the Flag peptide were further affinity purified by anti-HA antibody-conjugated agarose, and the final elutes from the HA-beads with HA peptides were resolved on an 8% SDS-PAGE gel for silver staining or subjected to mass spectrometric analysis (by MS Bioworks LLC).

### Immunoprecipitation and western blotting

Cells were washed twice with PBS, and then lysed in BC100 lysis buffer supplemented with protease inhibitor cocktail and phosphatase inhibitors (Sigma) for 1 h on ice with occasional vortex. Cell lysates were cleared by centrifugation at 14000 rpm for 20 min at 4 °C. For IP of Flag-tagged proteins, cell lysates with or without Flag-tagged proteins were incubated with anti-Flag M2 beads (Sigma) overnight at 4 °C. Beads were washed five times in lysis buffer and bound proteins were eluted with 1% SDS or Flag peptides (Sigma) in lysis buffer. For direct western blotting, the cells were washed twice with PBS and lysis in BC100 buffer containing 0.5% SDS and protease inhibitor cocktail and phosphatase inhibitors and sonicated for 20 s on power setting 1 using a sonic dismembrator (Model 100, Fisher Scientific). After measuring protein concentration using the Bio-Rad protein assay, equal amounts of proteins were loaded and separated on a SDS-PAGE gel ranging from 8–12%, and transferred to 0.45 μM nitrocellulose membranes (GE healthcare). Transfer quality was verified by Ponceau staining, followed by blocking with 5% milk/TBST/0.5 h, followed by overnight incubation with primary antibody diluted in 5% milk or BSA. The membranes were washed and incubated for 1 h in horseradish peroxidase (HRP)-conjugated secondary antibody diluted 1:1000–1:10,000. Clarity western ECL substrate (Bio-Rad) and blue film (Crystalgen) were used to detect the proteins. Densitometry of Western blots was performed using ImageJ software (https://imagej.net/Fiji). Western blot images were prepared using Photoshop CS6 (https://www.adobe.com/products/photoshop.html).

### Chromatin fractionation

Cell fractionation was performed largely according to the protocol described in the previous study^69^. Cells from 10-cm dishes were washed with PBS twice, and suspended in 200 μl of Buffer A (10 mM HEPES pH 7.9, 10 mM KCl, 1.5 mM MgCl_2_, 0.34 M sucrose, 10% glycerol, 10 mM NaF, 1 mM DTT, protease inhibitor cocktail and phosphatase inhibitors), and Triton X-100 was added to 0.1%, then the cells were left on ice for 5 min. The cytoplasmic fractions were separated from the nuclei by centrifugation at 1300 *g* for 4 min at 4 °C, and the cytoplasmic fractions were further clarified by centrifugation at 20,000 *g* for 5 min. The nuclei were washed one time with Buffer A, and lysed in 100 μl of Buffer B (3 mM EDTA, 0.2 mM EGTA, 1 mM DTT, protease inhibitor cocktail and phosphatase inhibitors) on ice for 30 min. The soluble nuclear fractions were separated from the chromatin fractions by centrifugation at 1700 *g* for 4 min. The chromatin fractions were washed one time with Buffer B and centrifuged at 10,000 *g* for 1 min, and lysed in 100 μl of BC100 buffer with 0.5% Triton and 0.5% SDS with sonication.

### GST pull-down assay

Recombinant proteins of GST-RAD17 full-length and different fragments, or of GST-SMG7 different fragments were expressed in Rosetta 2(DE3) cells (EMD, San Diego, CA, USA) and bound to glutathione agarose (Pierce, Rockford, lL, USA). GST-proteins beads were incubated with either purified proteins or cell lysates overnight at 4 °C and washed five times in BC100 washing buffer (20 mM Tris.Cl pH 7.3, 100 mM NaCl, 10% glycerol, 0.2 mM EDTA, 0.4% Triton X-100) supplemented with protease inhibitor cocktail. Bound materials were eluted with 2 × SDS-PAGE sample buffer and resolved by SDS-PAGE, transferred to nitrocellulose membranes, stained with ponceau for GST-proteins, and analyzed by western blotting.

### Silver staining

Proteins were separated on a polyacrylamide gel using electrophoresis. The gel fixed with 45% methanol/10% acetic acid for 30 min, followed by fixation with 10% glutaraldehyde for 10 min, thorough washing in water for 2 h, and treatment with 5ug/mL DTT for 30 min. The gel was incubated with 0.1% silver nitrate for 30 min, and quickly rinsed with water followed by carbonate developing solution. The gel was developed with carbonate developing solution, and the reaction was stopped using 5 mL of 2.3 M citric acid per 100 mL developing solution.

### Immunofluorescence

Cells were plated and grown on coverslips in 12-well plates for 20–24 h. For visualization of DNA damage foci, cells were treated with IR or CPT followed by pre-extraction with CSK buffer (10 mM Pipes pH 6.8, 100 mM NaCl, 300 mM sucrose, 3 mM MgCl_2_, 1 mM EGTA) with 0.7% Triton X-100 plus protein inhibitor cocktail for 5 min on ice. After washing once with CSK buffer without Triton X-100 and twice with PBS, cells were fixed (4% paraformaldehyde, 15 min, room temperature) and permeabilized (0.7% Triton X-100 in PBS, 10 min), blocked (5% BSA in PBS, 1 h), and incubated with primary antibody overnight. After extended wash in PBS-Triton buffer, incubated with fluorophore-conjugated secondary antibodies. After counterstaining with DAPI, coverslips were washed briefly with water and mounted in Prolong Diamond Antifade mounting media (#P36961, Invitrogen). Cells were visualized under an Olympus BX61 upright microscope using a 40 × oil objective. DNA damage foci were analyzed using ImageJ software and were defined by positive staining of distinct yH2AX or chromatin-bound RPA nuclear puncta.

Cell number and fluorescence intensity for RPA32/p-RPA32 S4/8 staining was quantified using the MetaMorph Multi-Wavelength Cell Scoring Application (https://www.moleculardevices.com/products/cellular-imaging-systems/acquisition-and-analysis-software/metamorph-microscopy) using cells pooled from three independent experiments. Cells were imaged using a 20 × air objective. Quantitation of RPA32-pS4/8 positive cells was performed as follows: DAPI staining was used to define the region of the nucleus of each cell, and this mask was used to measure the fluorescence intensity of RPA-pS4/8 in the TxRed channel. Untreated cells were used to establish the level of background fluorescence. Cells above this threshold were defined as RPA-pS4/8-positive, and cells below this threshold were defined as RPA-pS4/8-negative.

For BrdU detection, cells grown on coverslips were pulse labeled with 25 μM BrdU for 20 min and then washed with ice-cold PBS, fixed with 70% ethanol for 5 min and methanol (-20 °C) for 5 min and stored at 4 °C. DNA was denatured with 2.5 N HCl for 30 min and wash in PBS for 30 min. After blocking in blocking buffer (5% BSA in PBS with 0.5% tween-20) for 1 h, incubated with rat anti-BrdU (#MCA2060, Bio-Rad, 1:200) for 1 h, following washing in PBS and blocking buffer, incubated with fluorophore-conjugated anti-rat secondary antibody (#A11006, Invitrogen, 1:1000). DNA were counterstained with DAPI. Cells were visualized under an Olympus BX61 upright microscope using a 20 × air objective. Fluorescence intensity was quantified using the MetaMorph Multi-Wavelength Cell Scoring Application using cells pooled from three independent experiments. Data were analyzed as follows: DAPI staining was used to define the region of the nucleus. Unstained cells were used to establish the level of background fluorescence, and BrdU-stained cells above this threshold were defined as BrdU-positive. Using the mask created by DAPI, the fluorescence intensity of BrdU (of BrdU-positive cells) was measured in the FITC channel.

For BrdU/p-H3 S10 staining, cells were plated in log phase on poly-L-lysine-coated glass coverslips. Cells were pulsed with 25 μM BrdU for 30 min and treated with 5 mM hydroxyurea for 6 h. After treatment, the cells were washed and allowed to recover in fresh media in the presence of 1 μg/mL nocodazole. The cells were fixed with 2% PFA/10 min at 4 °C and permeabilized with 100% methanol/5 min at -20 °C. The cells were treated with 3 M HCl/30 min to denature the DNA and blocked with 2% BSA/0.5% Triton-X-100 before overnight incubation with primary antibodies (1:1000 rabbit anti-pH3-S10 (Santa Cruz #sc-8656); 1:200 rat anti-BrdU (clone BU1/75 (ICR1); AbD Serotec #MCA2060)) and 2-h incubation with secondary antibodies conjugated to AlexaFluor-488 and AlexaFluor-568. The cells were stained with DAPI and mounted in ProLong Diamond Antifade (Molecular Probes) before they were imaged using a 20 × air objective on a Olympus BX61 upright microscope. Cell number and fluorescence intensity were quantified using the MetaMorph Multi-Wavelength Cell Scoring Application using cells pooled from three independent experiments. Data were analyzed and graphs were generated using GraphPad Prism (https://www.graphpad.com/scientific-software/prism/). Fluorescent images were prepared using Photoshop CS6 (https://www.adobe.com/products/photoshop.html).

### Flow cytometry cell cycle analysis

Cells were plated and grown in 12-well plates overnight to log phase. Cells were pulsed with 25 μM BrdU for 20 min at 37 °C. The cells were washed with PBS × 2 and treated with media containing 5 mM hydroxyurea (Sigma-Aldrich) for 6 h at 37 °C. The HU was removed, the cells were washed with PBS × 2, and fresh media was added to allow recovery for 0, 4, 6, 8, and 12 h. After recovery, the cells were washed with PBS and harvested using 0.25% trypsin. The cells were fixed with 70% ethanol at -20 °C, pelleted at 1000 *g*, and rehydrated with 2% BSA/0.1% Tween20/PBS. The DNA was denatured with 3 M HCl for 30 min/RT, and the cells were blocked with 2% BSA/0.1% Tween20/PBS for 30 min/RT. BrdU was stained using a rat anti-BrdU antibody (1:200 rat α-BrdU (clone BU1/75; #MCA2060 BioRad)) for 1 h/RT, followed by incubation with 1:500 goat α-rat AlexaFluor-488 (Life Technologies) in the presence of RNAse for 2 h/RT. Equal numbers of cells were stained with 7-AAD (5ug/mL) and analyzed using a Becton Dickinson FACSCalibur flow cytometer and FlowJo software (https://www.flowjo.com/).

### DNA fiber assay

Cells were plated in log phase in 24-well plates. The cells were pulsed with media containing 25 μM 5-chloro-2′-deoxyuridine (CldU) for 30 min at 37 °C. The media was then removed, and the cells were pulsed with 250 μM iododeoxyuridine (IdU) + /- 2.5 μMcamptothecin (CPT) for 30 min at 37 °C. The cells were washed with PBS, harvested with 0.25% trypsin, and suspended in PBS to a concentration of 200,000 cells/mL. 2 μL cells were plated on a glass slide and allowed to dry slightly. The cells were lysed with 10 μL spreading buffer (200 mM Tris pH 7.4, 50 mM EDTA, 0.5% SDS) for 3 min, and the slides were tilted at a 20–30° angle to allow the DNA to spread. Excess lysate was blotted off the bottom edge of the slide, and the slides were allowed to dry for 1 h at room temperature. The DNA was fixed with 3:1 methanol/acetic acid for 3 min at − 20 °C. The DNA was denatured with 2.5 M HCl for 60 min at room temperature. The slides were blocked with 2% BSA/0.1% Tween20/PBS, and incubated with a-BrdU antibodies specific to CldU and IdU (1:100 rat α-BrdU (clone BU1/75; #MCA2060 BioRad), 1:100 ms α-BrdU (clone B44; #347,580 BD Biosciences)) and secondary (1:100 goat α-rat AlexaFluor 488; 1:100 goat α-ms AlexaFluor 568 (Life Technologies)) antibodies in a humidified chamber at 37 °C with washes in 0.1% Tween20/PBS in between. The slides were mounted with Prolong Diamond Antifade (Molecular Probes) and imaged with a 40 × air objective using an Olympus BX61 upright microscope. Data were analyzed using ImageJ software using fibers pooled from three independent experiments. Fork speed was calculated using the estimation that 1 µm corresponds to 2.6 kb as reported in a previous study^70^.

### Statistical analysis

Data were analyzed using Student’s t-test and one-way ANOVA with Tukey post-test using GraphPad Prism software (https://www.graphpad.com/scientific-software/prism/) and Microsoft Excel (https://www.microsoft.com/en-us/microsoft-365/excel). *** indicates *P* < 0.001, * indicates P < 0.05, n.s. indicates not significant (*P* > 0.05).

## Supplementary Information

Supplementary Information
