# Supplementary material for: Critical role of SMG7 in activation of the ATR-CHK1 axis in response to genotoxic stress
Source: Sci Rep. 2021 Apr 5;11:7502. doi: 10.1038/s41598-021-86957-x (PMC8021557; doi:10.1038/s41598-021-86957-x)
Supplement: Supplementary file 1 — Supplementary Information [file 41598_2021_86957_MOESM1_ESM.pdf]

Supplementary Figure 1

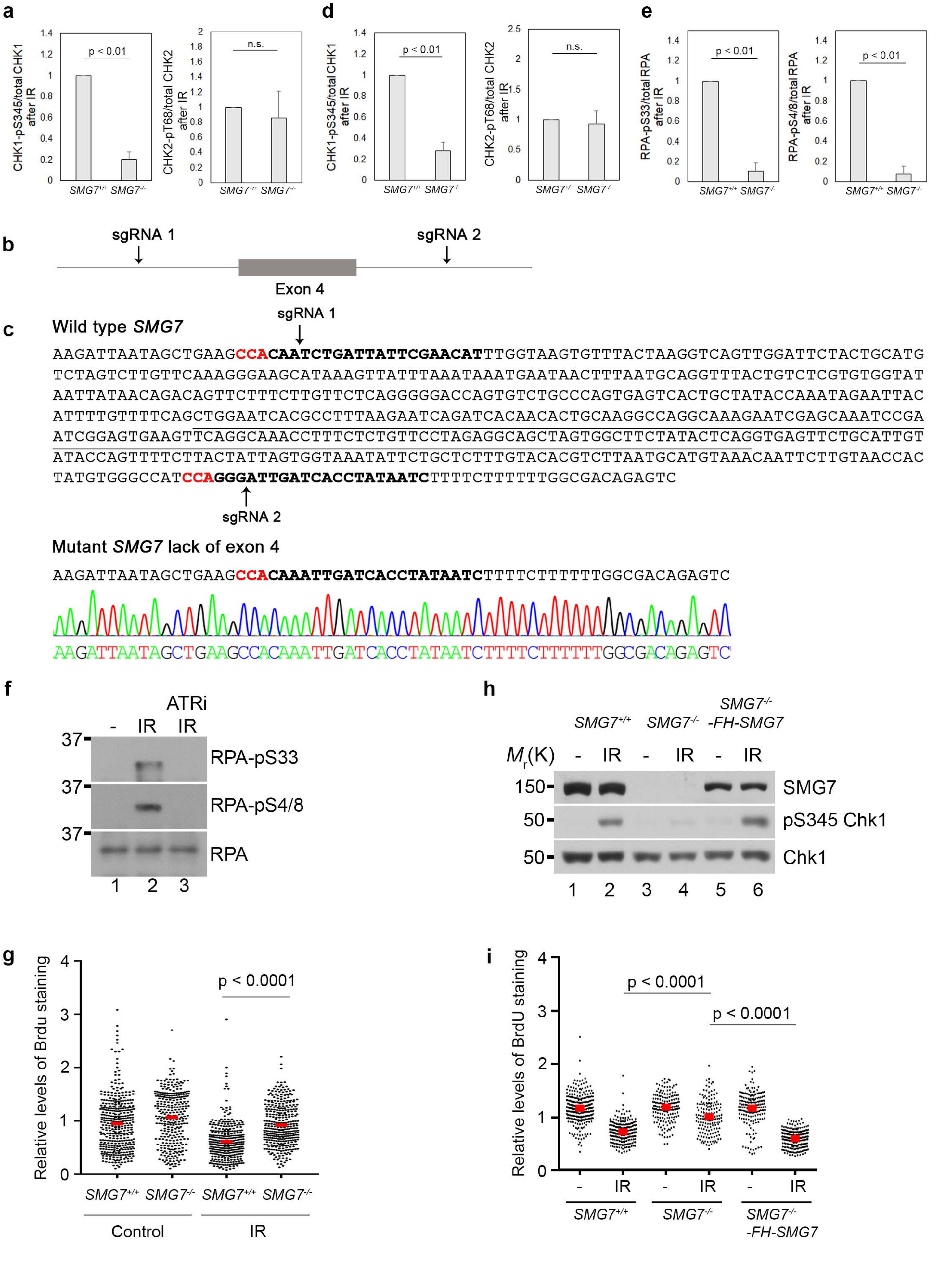

Supplementary Figure S1

a. Quantification of western blots from **Fig. 1a**. Levels of CHK1-pS345 and CHK2-pT68 in WT and SMG7$^{-/-}$ HCT116 cells after IR treatment were normalized to total CHK1 and CHK2 levels, and CHK1-pS345/total CHK1 and CHK2-pT68/total CHK2 in WT cells was set to 1. Relative levels of CHK1-pS345 (left) and CHK2-pT68 (right) after IR treatment are shown. Data are presented as Mean + SD (n=3 independent experiments) and were analyzed by Student's t-test (*P* values shown; n.s. not significant, $P > 0.05$).

b-c. CRISPR/Cas9-mediated gene targeting of *SMG7*. **b**. Two sgRNAs 1 and 2 flanking the *SMG7* exon 4, **c**. DNA sequences of wild type and mutant exon 4 region following gene targeting. Arrows show the Cas9 cutting sites.

d-e. Quantification of western blots from **Fig. 1b-c**. Levels of phosphorylated CHK1, CHK2, and RPA in WT and SMG7$^{-/-}$ cells after IR treatment were measured and normalized to total CHK1, CHK2, and RPA levels, and phosphorylated/total protein in WT cells was set to 1. Data are presented as Mean + SD (n=3 independent experiments) and were analyzed by Student's t-test (*P* values shown; n.s. not significant, $P > 0.05$). **d.** Quantification of Western blots from **Fig. 1b**. Relative levels of CHK1-pS345 (*left*) and CHK2-pT68 (*right*) in WT and SMG7$^{-/-}$ DLD1 cells treated with 10 Gy ionizing radiation. **e.** Quantification of Western blots from **Fig. 1c.** Relative levels of RPA-pS33 (*right*) and RPA-pS4/8 (*left*) in DLD1 cells treated with 20 Gy ionizing radiation are shown.

f. Wild type DLD1 cells were treated with ionizing radiation (20 Gy/2 hrs) +/- ATRi (10μm VE-822) and total cell extracts were examined for RPA-pS33 and RPA-pS4/8.

g. Wild type and *SMG7*$^{-/-}$ DLD1 cells were treated with ionizing radiation (10Gy), and 0.5 hour later labeled with 25uM BrdU, and analyzed by immunostaining using α-BrdU antibody (BU1/75). The relative intensity of BrdU staining was quantified in BrdU+ cells. Red bars

represent the mean intensities of each BrdU+ population. Data were analyzed by ANOVA with Tukey post-test. *P* values are shown.

h. Wild type, *SMG7*$^{-/-}$ DLD1 cells, and *SMG7*$^{-/-}$ DLD1 cells expressing full-length FLAG-HA-SMG7 (SMG7$^{-/-}$-FH-SMG7) were treated with ionizing radiation (10Gy/0.5 hr). Total cell extracts were examined for CHK1-pS345.

i. Wild type, *SMG7*$^{-/-}$ DLD1 cells, and *SMG7*$^{-/-}$ DLD1 cells expressing full-length FLAG-HA-SMG7 (SMG7$^{-/-}$-FH-SMG7) were treated with ionizing radiation (10Gy), and 0.5 hour later labeled with 25uM BrdU. The relative intensity of BrdU staining was quantified in BrdU+ cells. Red bars represent the mean intensities of each BrdU+ population. Data were analyzed by ANOVA with Tukey post-test. *P* values are shown.

Supplementary Figure 2

a

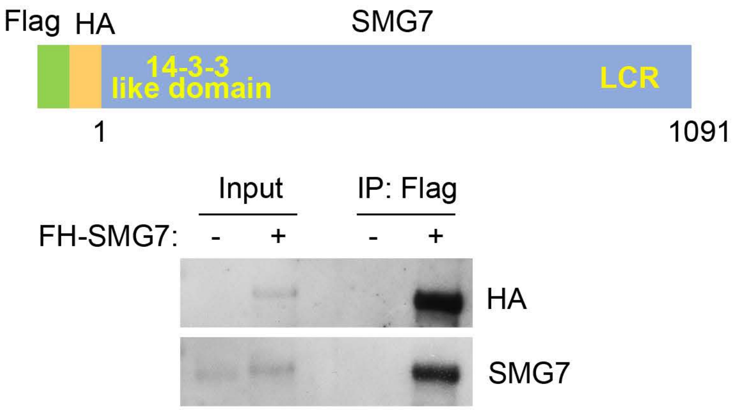

b

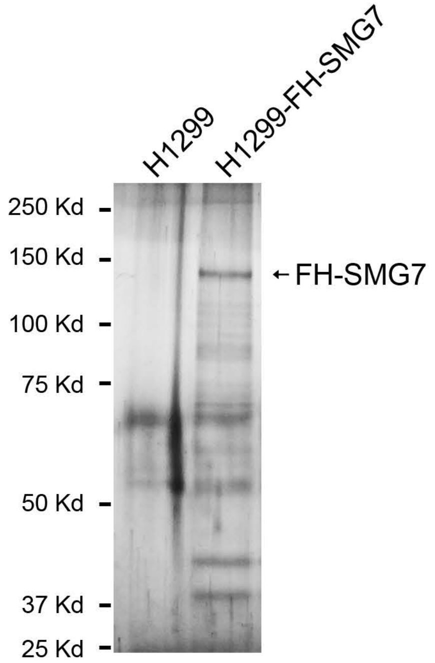

d

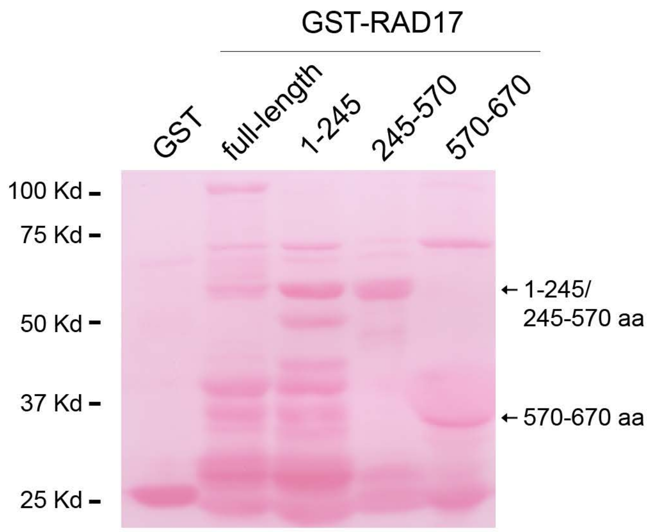

c

Protein	Number of peptides
SMG7	540
UPF1	119
SMG5	66
RFC3	17
RAD17	15
RFC4	14
RFC2	11
RFC5	8

RAD17 peptides identified by Mass spectrometry

(51) NGPSTLESSR(60)
(128)QGGSILLITGPPGCGK(143)
(235)DSHTLHEVLR(244)
(335)SAINSLQFSSSK(346)
(378)VFENQEVQAIGGK(390)
(492)EYSTSIATR(500)
(596)LKMEALTDR(604)

RFC protein peptides identified by Mass spectrometry

RFC2
(47) LNEIVGNEDTVSR(59)
(66) EGNVPNIIIAGPPGTGK(82)
(167)FALACNASDK(176)
(193)LTDAQILTR(201)
(202)LMNVIEK(208)
(254)VCDEPHPLLVK(264)

RFC3
(16) LDYHKEQAAQLR(27)
(58) ELYGVGVEK(66)
(69) IEHQTITTPSK(79)
(106)VVIQEMLK(113)
(114)TVAQSQQLETNSQR(127)
(131)VVLLTEVDKLTK(142)
(143)DAQHALRR(150)
(226)ALLMCEACR(234)
(257)ETANAIVSQQTPQR(270)

RFC4
(7) GTSISTKPPLTK(18)
(105)VLELNASDER(114)
(136)SDGKPCPPFK(145)
(186)IIEPLTSR(193)
(211)LLDIAKK(217)
(222)ISDEGIAYLVK(232)
(240)KAITFLQSATR(250)

RFC5
(67) TSTILACAK(75)
(103)GPILSFASTR(112)
(204)ALVTLSSGDMRR(215)
(216)ALNILQSTNMAFGK(229)
(312)LSVGTNEK(319)

Supplementary Figure S2

a. Schematic showing domain structure of Flag-HA-SMG7 fusion protein (top) and validation of Flag-HA-SMG7 stably-expressing H1299 cells (bottom). Immunoprecipitation was performed using anti-Flag M2 agarose beads, and cell extracts and immunoprecipitates were analyzed by western blot using α-HA and α-SMG7 antibodies.

b-c. Immunoprecipitated proteins following Flag-HA tandem purification from H1299-FH-SMG7 cells were separated by SDS-PAGE and visualized by silver staining in **b**. Proteins from the immunoprecipitates were identified by mass spectrometry in **c**. Peptides identified from selected proteins are shown.

d. GST, GST-RAD17 full-length and fragment fusion proteins used in the GST pull-down assay (**Fig. 2g**) were separated by SDS-PAGE and transferred to nitrocellulose membrane followed by staining with Ponceau red. Arrowheads indicate the location of each fragment.

Supplementary Figure 3

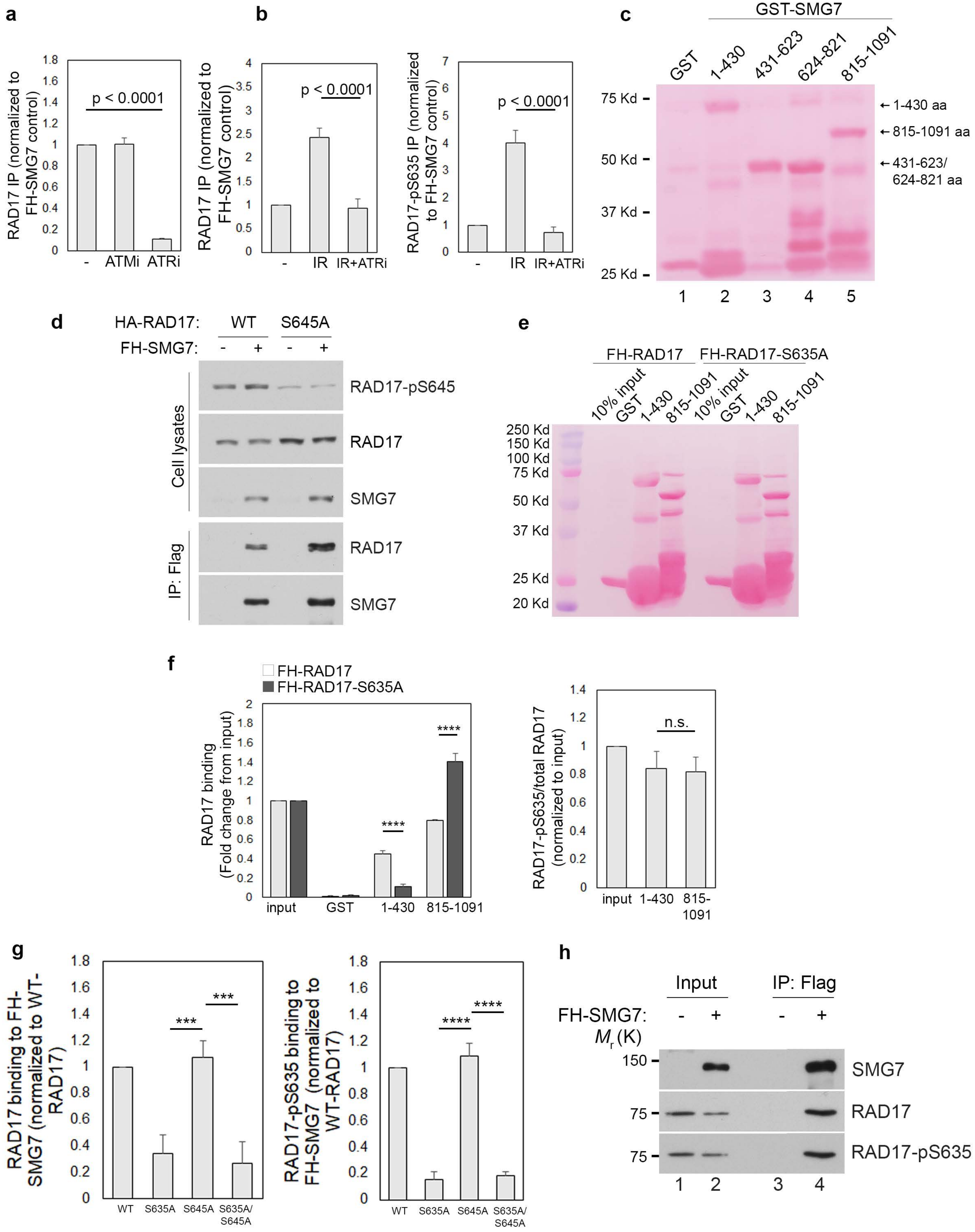

Supplementary Figure S3

a-b. Quantitation of western blots from **Fig. 3a-b**. Levels of immunoprecipitated RAD17 were normalized to total RAD17 in the input. Data were analyzed by one-way ANOVA with Tukey post-test and are presented as Mean + SD (n=3 independent experiments). *P* values are shown. **a.** Quantitation of western blot from **Fig. 3a**. Levels of RAD17 in ATMi- and ATRi-treated FH-SMG7-expressing cells were normalized to RAD17 levels in untreated FH-SMG7-expressing cells (lane 2 in western blot). **b**. Quantitation of western blot from **Fig. 3b**. Levels of RAD17 (*left*) and RAD17-pS635 (*right*) in FH-SMG7-expressing cells treated with IR and IR+ATRi were normalized to untreated FH-SMG7 cells (lane 2 in western blot).

c. GST and GST-SMG7 fragment fusion proteins used in the GST pull-down assay (see **Fig. 3c**) were visualized by SDS-PAGE followed by transfer to nitrocellulose and staining with Ponceau red. Arrowheads indicate the location of each fragment.

d. Interaction between SMG7 and RAD17 S645A mutant. H1299 cells were co-transfected with plasmids expressing FH-SMG7 and HA-RAD17 WT or S645A mutant. Cell extracts and α-Flag immunoprecipitates were analyzed by western blot using antibodies indicated.

e. Ponceau staining of GST and GST-SMG7 N- and C-terminal fragments used in **Fig. 3e**.

f. Quantitation of western blots from **Fig. 3e**. Data were analyzed by one-way ANOVA with Tukey post-test and are presented as Mean + SD (n=3 independent experiments; ****$P <$ 0.001, n.s. not significant, $P > 0.05$). (*Left*) Relative levels of RAD17 binding to SMG7 fragments in cells transfected with FH-RAD17 and FH-RAD17-S635A. Levels of RAD17 from the GST pull-down of SMG7 fragments were measured and normalized to levels of RAD17 in the input. (*Right*) Comparison of RAD17-pS635 binding to 1-430aa and 815-1091aa SMG7 fragments. Levels of RAD17-pS635 were measured and normalized to total RAD17, and RAD17-pS635/total RAD17 levels in the input was set to 1.

g. Quantitation of western blots from **Fig. 3f**. Relative binding of RAD17 (left) and RAD17-pS635 (right) to FH-SMG7 in RAD17 S635A, S645A, and S635A/S645A mutants. Levels of

immunoprecipitated RAD17 and RAD17-pS635 in cells transfected with WT-RAD17 were set to 1. Data are presented as Mean +SD (n=3 independent experiments; ***$P < 0.001$, ****$P < 0.0001$).

h. Characterization of levels of endogenous RAD17 binding to FH-SMG7. H1299 cells were transfected with FH-SMG7, and immunoprecipitated endogenous RAD17 and RAD17-pS635 were analyzed by western blot using the antibodies indicated.

Supplementary Figure 4

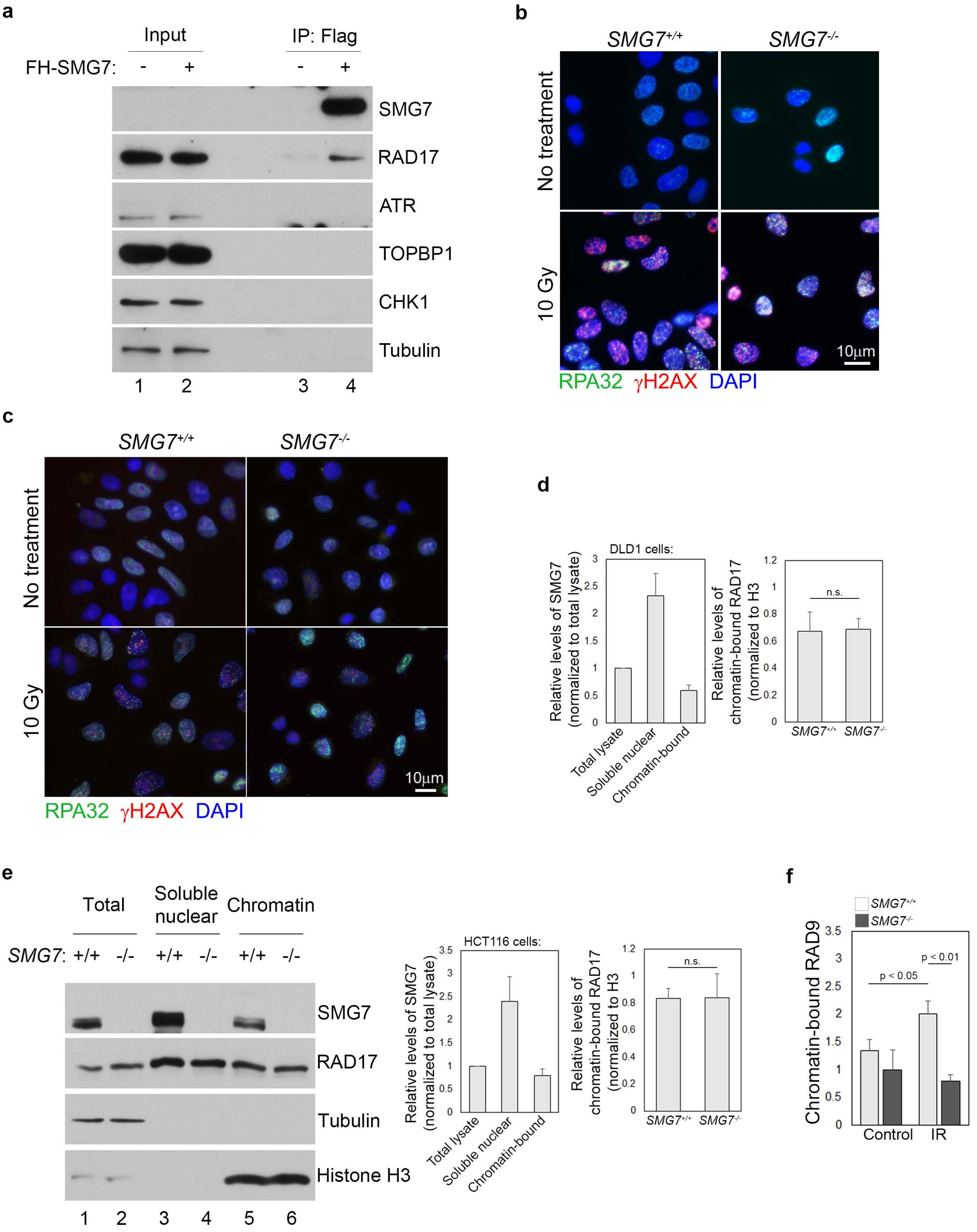

Supplementary Figure S4

a. Cell extracts from HCT116 *SMG7*$^{-/-}$ cells expressing FH-SMG7 and the anti-FLAG immunoprecipitates were analyzed by western blot.

b. Wider field of view of wild type and *SMG7*$^{-/-}$ DLD1 cells treated with 1μM CPT/1 hr and pre-extracted prior to fixation (See **Fig. 4b**). Cells were stained for RPA32 (green), γH2AX (red), and DAPI (blue) and imaged with a 40x objective. Merged representative images are shown.

c. Wider field of view of wild type and *SMG7*$^{-/-}$ cells treated with 10 Gy ionizing radiation for 1 hr and pre-extracted prior to fixation (See **Fig. 4c**). Cells were stained for RPA32 (green), RAD9 (red), and DAPI (blue) and imaged with a 40x objective. Merged representative images are shown.

d. Quantitation of western blots from **Fig. 4e**. The same amount of protein for each fraction was loaded in each well. (*Left*) Levels of SMG7 from the total cell lysate, soluble nuclear fraction, and chromatin bound-fraction of WT DLD1 cells were normalized to levels of SMG7 in the total lysate. (*Right*) Levels of RAD17 in the chromatin-bound fraction were measured and normalized to H3. Data are presented as Mean + SD (n=3 independent experiments; n.s. not significant, $P > 0.05$).

e. Wild type and *SMG7*$^{-/-}$ HCT116 cells were subjected to fractionation, and the total, soluble nuclear and chromatin fractions were analyzed by western blot. (*Left*) Levels of SMG7 from the total cell lysate, soluble nuclear fraction, and chromatin bound-fraction were normalized to levels of SMG7 in the total lysate. (*Right*) Levels of RAD17 in the chromatin-bound fraction were measured and normalized to H3 (*right*). Data are presented as Mean + SD (n=3 independent experiments; n.s. not significant, $P > 0.05$).

f. Quantitation of Western blots from **Fig. 4f**. Levels of chromatin-bound RAD9 were normalized to H3. Data were analyzed by one-way ANOVA with Tukey post-test and are presented as Mean + SD (n=3 independent experiments; *P* values are shown).

Supplementary Figure 5

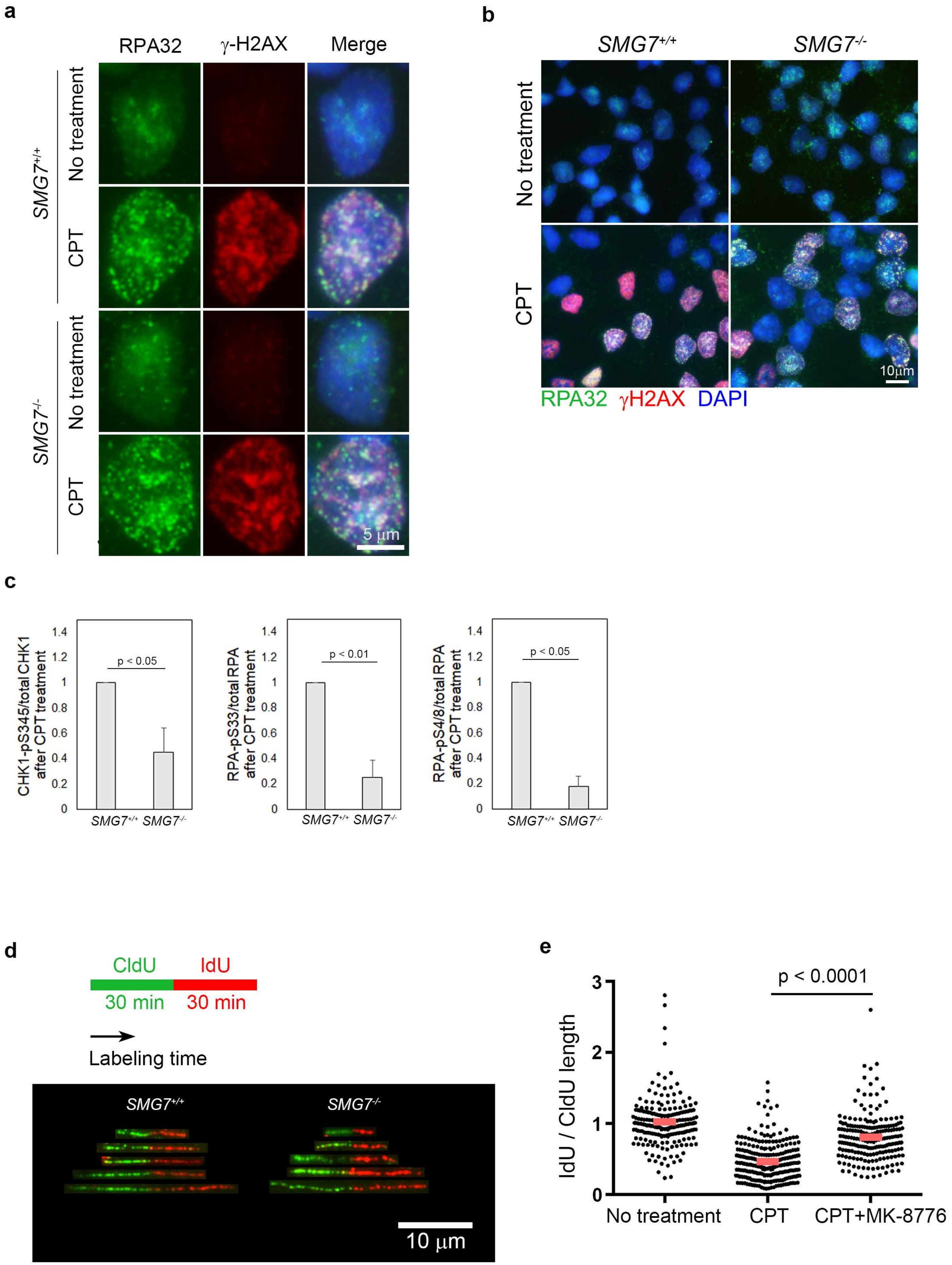

Supplementary Figure S5

a. Wild type and *SMG7*$^{-/-}$ HCT116 cells were treated with 1 μM CPT for 1 hour, followed by extraction of soluble nuclear proteins with CSK buffer/0.7% TX-100 and immunostaining with α-RPA32 (green) and α-γH2AX (red). Representative images were shown.

b. Wider field of wild type and *SMG7*$^{-/-}$ HCT116 cells treated with 1 μM CPT for 1 hour prior to pre-extraction (See **Fig. S5a**). Cells were imaged with a 40x objective and stained for RPA32 (green), γH2AX (red), and DAPI (blue). Merged representative images are shown.

c. Quantification of western blots from **Fig. 5a**. Relative levels of CHK1-pS345 (*left*), RPA-pS33 (*center*), and RPA-pS4/8 (*right*) in WT and SMG7$^{-/-}$ HCT116 cells after CPT treatment. Data are expressed as levels of CHK1-pS345, RPA-pS33, and RPA-pS4/8 normalized to total CHK1 and RPA. WT CPT-treated cells were set to 1. Data were analyzed by Student's t-test and are presented as Mean + SD (n=3 independent experiments). *P* values are shown.

d. DNA fiber analysis was performed using wild type and *SMG7*$^{-/-}$ HCT116 cells labeled with CldU and IdU. Labeling scheme (top) and representative images of DNA fibers (bottom) are shown.

e. DNA fiber analysis was performed using wild type HCT116 cells labeled with CldU and IdU. Cells were treated with CPT with or without CHK1 inhibitor (10μM MK-8776) concurrent with the IdU labeling. The lengths of IdU tracks and CldU tracks of ongoing replication forks (IdU-CldU-labeled tracts) were measured. Data is presented as the IdU:CldU length ratio. Red bars represent the mean of each population. Data were analyzed by one-way ANOVA with Tukey post-test. *P* values are shown.

Supplementary Figure 6

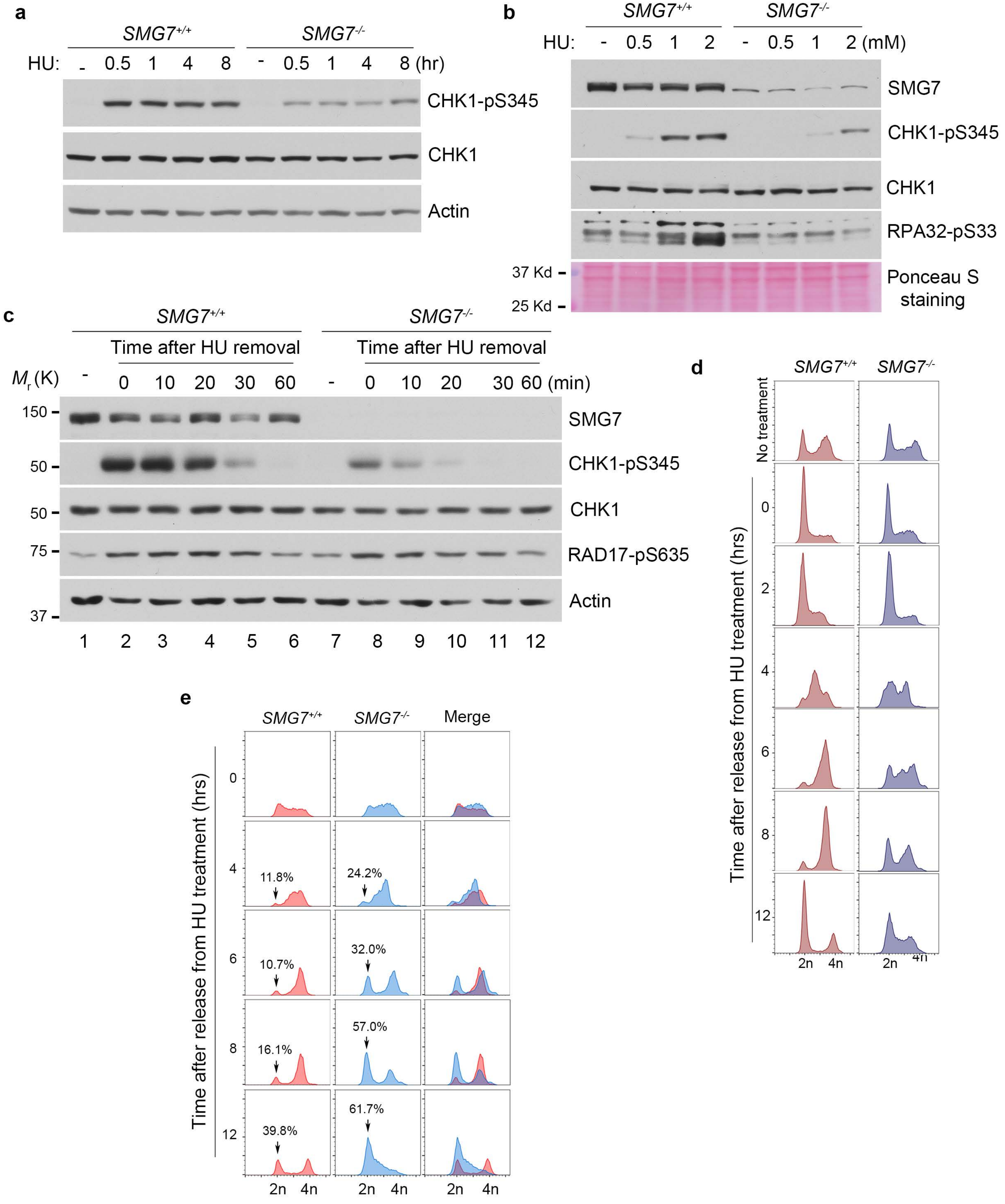

Supplementary Figure S6

a-b. Cell extracts from wild type and *SMG7*$^{-/-}$ HCT116 cells treated with HU were analyzed by western blot using α-SMG7, α-RPA32-pS33, α-CHK1-pS345, α-CHK1, and α-Actin antibodies. Ponceau staining indicates even loading of proteins. **a**. 0.5, 1 and 2 mM HU for 1 hour, **b**. 1 mM HU for 0.5, 1, 4 and 8 hrs.

c. Wild type and *SMG7*$^{-/-}$ HCT116 cells were treated with 1 mM hydroxyurea (HU) for 1 hour, followed by HU removal and release into fresh normal media. Cells were harvested at the indicated time points, and cell extracts were analyzed by western blot using α-SMG7, α-CHK1-pS345, α-CHK1, α-RAD17-pS635 and α–Actin antibodies.

d. Wild type and *SMG7*$^{-/-}$ HCT116 cells were treated with 5 mM HU for 6 hours and released into fresh normal media. Cells were fixed, stained with 7-AAD and analyzed by flow cytometry. 2n and 4n indicate the DNA content of cells in G_1 and G_2, respectively.

e. Cells treated as in **Fig. 6b** were gated based on BrdU staining, and the BrdU-positive cells were analyzed by flow cytometry (presented as histogram). Arrows indicate the cell population containing 2n DNA content.

Original Western Blots

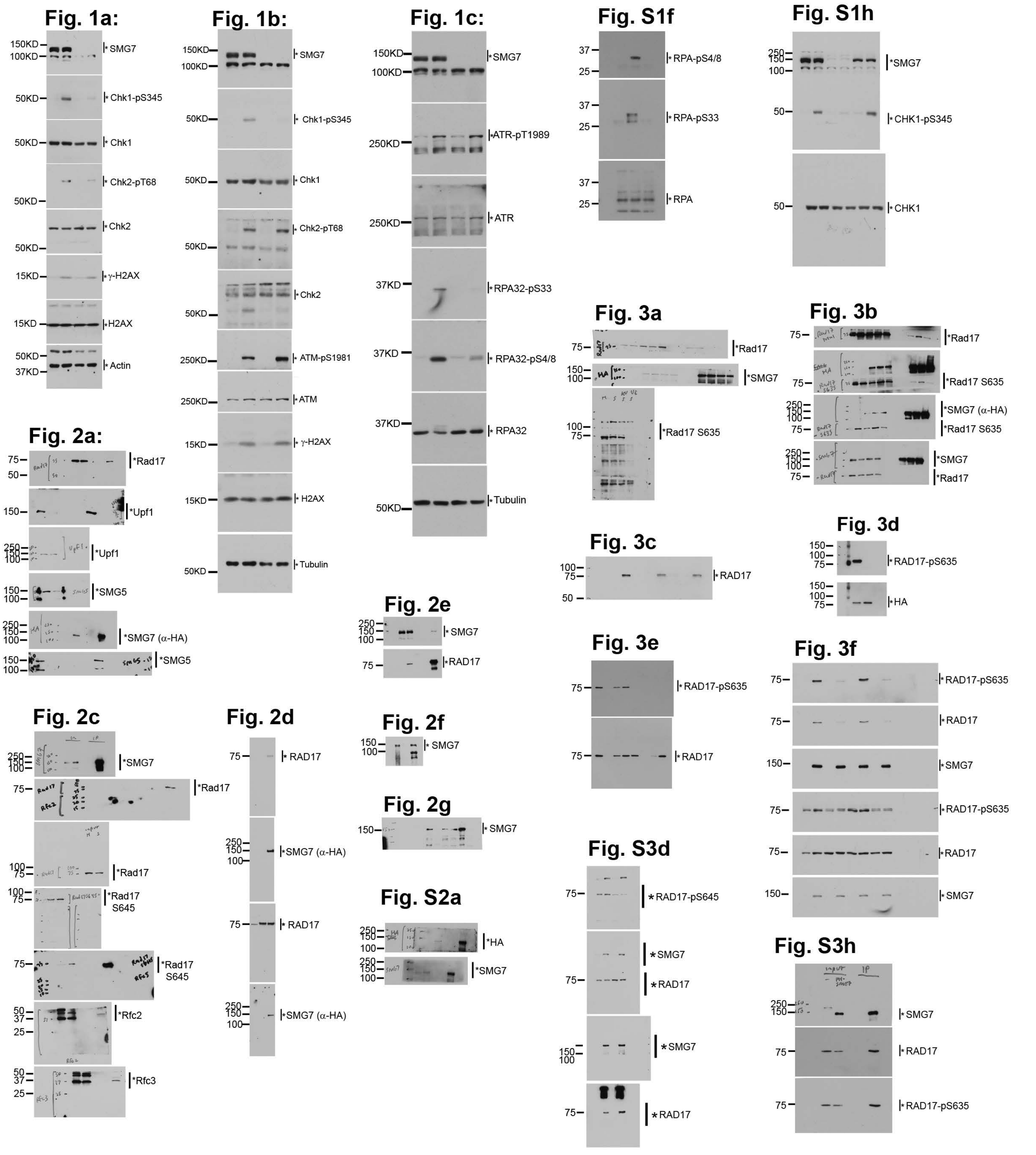

|* Region in Figure

Original Western Blots

Fig. 4a

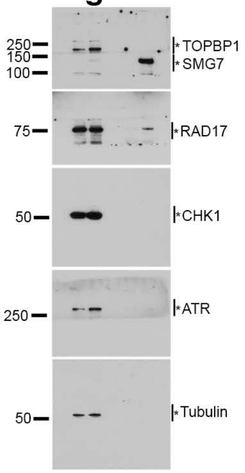

Fig. 4e

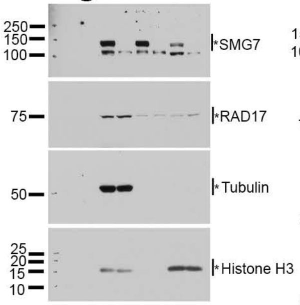

Fig. 4f

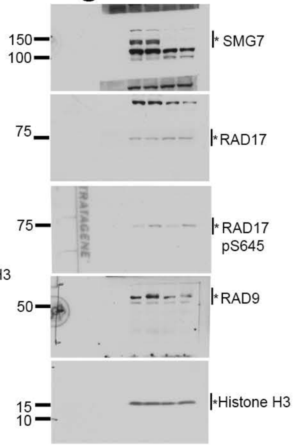

Fig. S4a

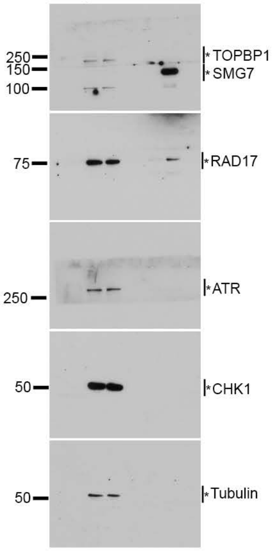

Fig. S4e

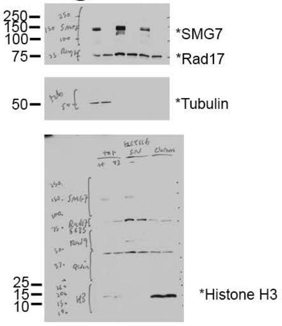

Fig. 5a

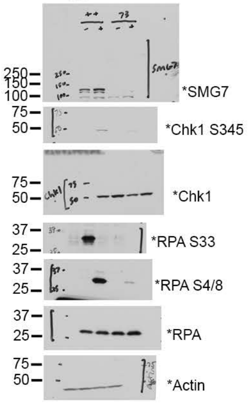

Fig. 6e

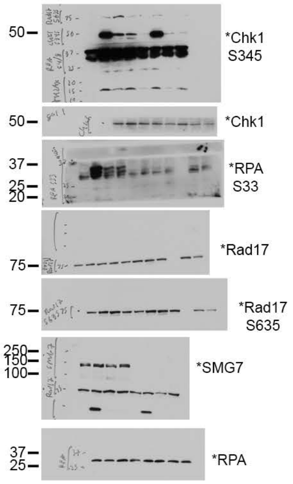

Fig. S6a

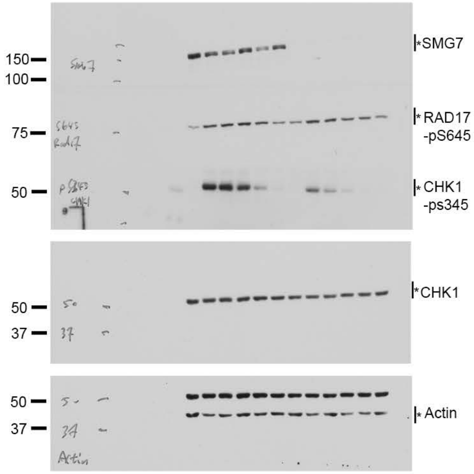

Fig. S6b

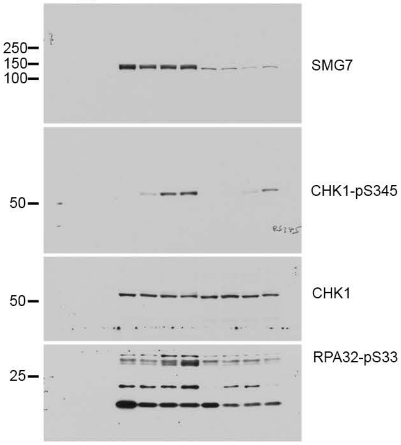

Fig. S6c

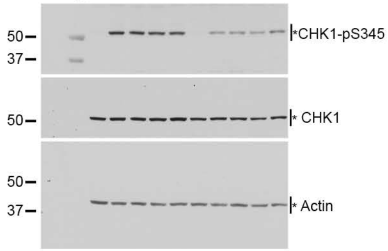

|* Region in Figure
